# The forest frontier in the Global South: Climate change policies and the promise of development and equity

**DOI:** 10.1007/s13280-021-01602-1

**Published:** 2021-09-03

**Authors:** Maria Brockhaus, Monica Di Gregorio, Houria Djoudi, Moira Moeliono, Thuy Thu Pham, Grace Y. Wong

**Affiliations:** 1 Department of Forest Science, Chair of International Forest Policy, University of Helsinki (Helsinki) and Helsinki Sustainability Center (HELSUS), Helsinki, Finland; 2 CIFOR, Bogor, Indonesia; 3 Research Institute for Humanity and Nature, Kyoto, Japan; 4 Sustainability Research Institute, School of Earth and Environment, University of Leeds, Leeds, UK; 5 CIFOR, Bogor, Indonesia; 6 Stockholm Resilience Centre, Stockholm University, Stockholm, Sweden

**Keywords:** Climate governance, Forest frontier, Inequality, Maladaptation, Politics, REDD+

## Abstract

Halting forest loss and achieving sustainable development in an equitable manner require state, non-state actors, and entire societies in the Global North and South to tackle deeply established patterns of inequality and power relations embedded in forest frontiers. Forest and climate governance in the Global South can provide an avenue for the transformational change needed—yet, does it? We analyse the politics and power in four cases of mitigation, adaptation, and development arenas. We use a political economy lens to explore the transformations taking place when climate policy meets specific forest frontiers in the Global South, where international, national and local institutions, interests, ideas, and information are at play. We argue that lasting and equitable outcomes will require a strong discursive shift within dominant institutions and among policy actors to redress policies that place responsibilities and burdens on local people in the Global South, while benefits from deforestation and maladaptation are taken elsewhere. What is missing is a shared transformational objective and priority to keep forests standing among all those involved from afar in the major forest frontiers in the tropics.

**Supplementary Information:**

The online version contains supplementary material available at 10.1007/s13280-021-01602-1.

## Introduction

Tropical forests and lands are highly visible on today's political agendas and are being claimed for a myriad of global, national, and local interests linked to timber, biomass resources, and the production of commodities such as soy, oil palm, and pulp and paper. They are also the scene of ‘sustainable’ and low emission development, poverty reduction, conservation, and ‘green’ growth (Redclift 1997; Scheidel and Sorman 2012; Seymour and Busch 2016). These often conflicting interests and ideas shape forest lands in the Global South as locations where natural environments are turned into resource and commodity frontiers (Kroeger and Nygren 2020). Here, government authorities, private sector actors, conservationists, communities, environmental defenders, and other members of civil society execute their agency and negotiate divergent interests. Yet, there are power imbalances among these actors, often to the disadvantage of local people and environments (Curtis et al. 2018; Peluso and Vandergeest 2020). Under these conditions, inequality is reinforced, produced, and reproduced in the access to and benefits from these forest lands in the Global South. Never the result of single, distinct factors but the outcome of intersections of different social locations, power relations, and experiences (Hankivsky 2014), inequality is both part of the local and global processes and outcomes (Newell 2005).

Forest-based climate change adaptation and mitigation are the most recent additions to this long list of interests and ideas over forests and forest lands in the Global South, with carbon and non-carbon benefits as tangible and intangible commodities. Within the literature, there is concern that with the implementation of new forest and climate governance tools, unsustainable exploitation and associated inequalities will simply continue or even be aggravated (Lund et al. 2017; Dawson et al. 2018), despite ambitions and commitments to the contrary (see, for example, the New York Declaration of Forests, the Sustainable Development Goals, and the Paris Agreement). As scholars argue, political transformational change is required for forest and climate governance to break with a history of deforestation, failed adaptation, and unequal development (Brockhaus and Angelsen 2012; Scoones et al. 2015; Temper et al. 2018; Martin et al. 2020). In the context of this paper, we define transformational change as shifts in power relations, discursive practices, and incentive structures that lead away from unsustainable and unjust exploitation in forest frontiers in the Global South (Brockhaus and Angelsen 2012). Examples of transformational change would include changes of the larger social, economic, and regulatory frameworks that govern forests and forest lands, changing global trade and investment patterns, removals of subsidies, and other perverse incentives fueling exploitation, as well as forest industry and sector-specific reforms. At the same time, we observe the persistence of an unsustainable and often unjust business-as-usual (BAU) practice of forest land exploitation. In this paper, we ask what enables and what hinders efforts to break this BAU. The key question we explore is if and how climate governance can positively affect these threatened forest frontiers and facilitate socially and environmentally just transitions away from BAU.

We explore these questions by taking a comparative and multi-level case study approach. The four cases are based on the authors’ research conducted over the past two decades linked to four distinct forest and climate change adaptation and mitigation studies. They consist of observations from Southeast Asia, South America, and West Africa and are situated in different temporal, spatial, and socio-economic intersections of forest, climate change, and economic development in the Global South. We adopt a political economy lens to unpack processes of change along the forest and climate change frontier and the embedded processes of resource control and extraction and commodity production. The comparative approach allows us to uncover patterns of business-as-usual and transformational change across the diverse climate and forest frontiers.

The paper is organised as follows. First, we introduce our framework and the 4Is (Institutions, Interests, Ideas, and Information), which we use to examine each of the cases. The discussion builds on the comparative analysis of the cases and identifies power and politics structures that are useful to explain shifts towards transformational change, as well as the lack thereof, namely the persistence of BAU outcomes in global forest governance. We close the paper with a reflection on possible pathways for change.

## Background and theoretical framework

Forest degradation and deforestation in the tropics pose a major challenge to climate change adaptation and mitigation efforts (IPCC 2007). Yet, the underlying problem definition and proposed solutions to this wicked problem are often guided by so-called ‘myths’ in global forest governance (Delabre et al. 2020). A prominent and persistent myth is the assumption that states and government bureaucracies manage the forest autonomously from large-scale economic interests driving deforestation, with an intention to achieve what is best for their country’s society. This assumed autonomy of state actors has been questioned for the case of REDD+ in an investigation of the politics of deforestation in the tropics (Di Gregorio et al. 2012). Another popular myth is related to smallholders and the promise that ‘participation’ in global forest governance will solve deforestation, which ignores power imbalances and implies that local people’s land-use practices are the main cause of the problem (Skutsch and Turnhout 2020). Mobility in land use, in the form of shifting cultivation practices and pastoralism, for example, are such ‘problematised’ practices, justifying efforts to stop what some scholars point out are highly adaptive and sustainable land-use systems in areas with high soil and climate variability (Turner 2011; Djoudi et al. 2013; Bruun et al. 2018; Liao et al. 2020). In recent decades, local environmental activists protesting dispossession set in motion numerous initiatives to halt deforestation and forest conversion at grassroots levels. In parallel, national policies and international programmes to halt tropical deforestation multiplied. However, many of those defending their forests have lost their lives at the hands of business-as-usual interests (Global Witness 2019; Scheidel et al. 2020), and deforestation in the tropics with loss of old-growth forest continues at high rates (Curtis et al. 2018; Harris et al. 2021). Earlier declines in forest loss in Brazil were followed by a very sharp increase in deforestation, accompanied by increasing levels of ‘perverse’ incentives for activities such as biofuel production in the Amazon largely at the expense of old-growth forests (Ferrante and Fearnside 2020). For global forest governance to foster sustainability in tropical landscapes, those attempting to halt deforestation and enabling local forest-based adaptation will need to recognise the power dynamics and complex interactions resulting in injustices and inequalities within and across communities, societies, and regions (Locatelli et al. 2008; Menton et al. 2020).

**Fig. 1:**
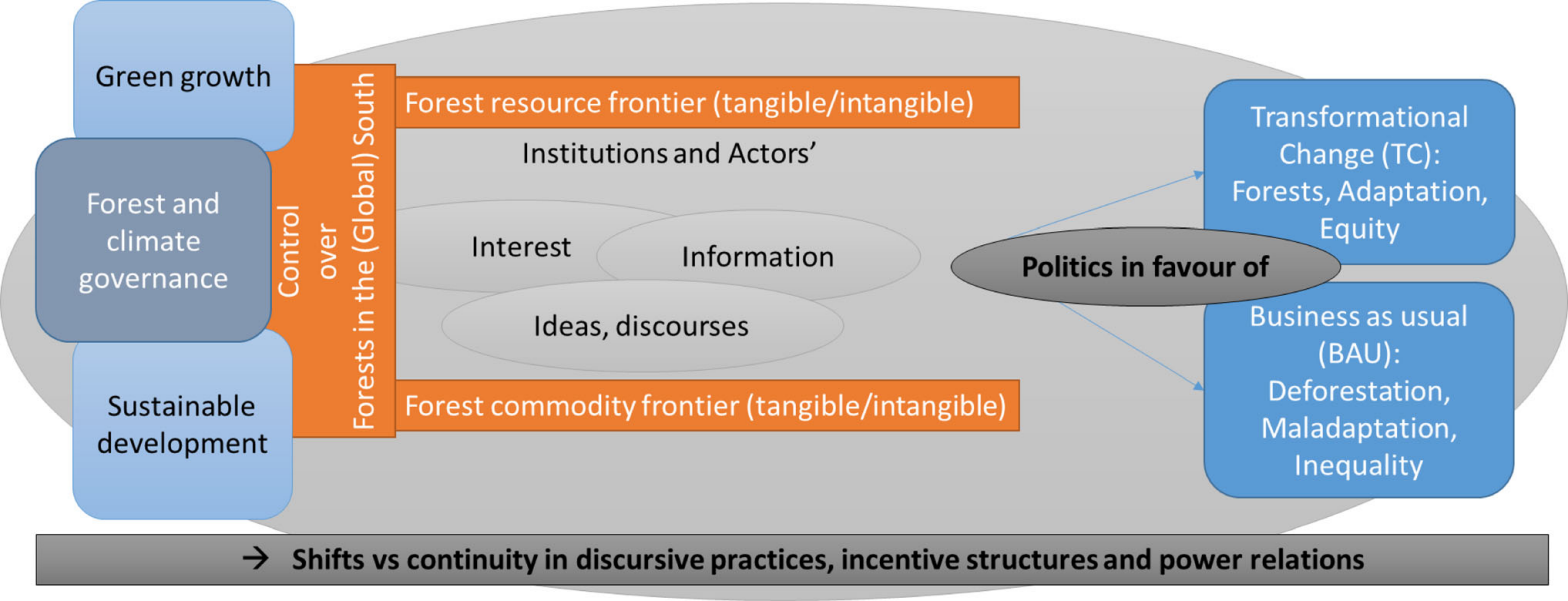
Politics for change or business as usual (BAU): Local forest frontiers, global calls for climate change policies, and the glocal 4Is (institutions, interests, ideas, and information)

Against this backdrop, a political economy in forest and lands in the Global South through the establishment of resource and commodity frontiers becomes visible, with **Institutional** path dependencies created and reinforced by and affecting diverse actors at diverse levels in pursuit of their **Interests**, favoured or marginalised by specific **Ideas** and myths and further honed by available or lacking **Information** and transparency; what we call the ‘4Is’ (Brockhaus and Angelsen 2012). In our conceptualisation of the forest frontiers, we draw attention to sites in the Global South where climate policy efforts, often combined with promises of sustainable development, green growth, and prosperity meet with powerful BAU interests within already established resource extraction and commodity production frontiers. These assemblages contribute to the construction of new tangible and intangible global resources and commodities, while subsumed within (neo)colonial discourses and legal frameworks. Outcomes of these processes might contribute to global as well as national inequalities, where high-consumption lifestyle demands of the global North as well as those of powerful elites within the country are sustained, while neglecting the livelihood needs of local people not being part of these elites and leaving behind societies in developing countries in terms of achieving progress with the SDGs (Redclift and Sage 1998; Xu et al. 2020). These frontiers are not politics-free spaces, shaped by an imposed frontier governance. Rather, they can be understood as a (forest) governance frontier (Thaler et al. 2019), in recognition of the role of politics in constructing and transforming frontier spaces and in resisting these transformations. Underlying power structures shape ideational and economic accounts of frontier development linked to control, resource extraction and commodity production. We add to this conceptualisation by further unpacking power and politics in tropical forest frontiers under climate change through a comparative perspective and applying the 4Is framework introduced above to extract shared and differentiated conditions enabling change and/or the continuation of business as usual. In our efforts to avoid an easily over-simplified dichotomy of North–South interests that risks ignoring local agency in the processes of change, and the role of China and other South–South relations in many of our forest frontiers, we pay particular attention to the dynamics and interactions among different international, national, and local actors over time, the inequalities in this process and the outcomes, and the diversity of benefits and burdens generated in each of the following cases. We investigate to what extent we see shifts in power and discursive practices, and incentive structures with the introduction of climate change policies and programmes in the frontiers, what elements of BAU continue to remain across cases and what this means for sustainable and fair forest frontiers (Fig. 1).

## Materials and Methods

For the purpose of this paper, the authors reviewed their earlier work and case material collected over recent decade(s) in specific research projects related to forests and climate change in the Global South (see Table 1). Detailed descriptions of the cases can be found in the Supplementary information to this paper. The mix of methods used varied across the project-based cases, with mostly qualitative data from interviews, focus group discussions (FGDs), and workshops and policy documents collected and analysed. We conducted disaggregated FGDs (by gender and age in Case 2, and additionally by ethnicity in Case 3) at local levels, and workshops/FGDs at regional and national levels on the intersection of forest, climate, and development policies. For case 3 on forests and adaptation, we used participatory methods to understand perceptions and priorities related to the forests and climate change governance across levels of governance. Policy document analysis took place in all cases, mainly based on deductive and inductive coding applied through a critical discourse or institutional theory lens. Cases 1, 2, and 4 also used surveys that allowed for quantitative analysis of actors’ position statements, of coalition work, and of policy network structures in the REDD+ and wider land-use policy arenas. Case 2 also utilised social network analysis. Case 1 included longitudinal research, and we repeated the network analysis twice in six countries and completed three rounds of a qualitative comparative analysis (QCA) based on expert assessments in 16 REDD+ countries. In addition, we also conducted a systematic media analysis across nine countries to investigate which actors put forward specific views and positions towards forest-based climate mitigation. For the purpose of this paper, we complemented these case-specific analyses with a review of the wider literature on forest frontiers and transformational change (see Table 1 on methods).

**Table 1:** Cases, authors, research projects, and methods applied in different cases

Case	Brief description	Methods	Geographical scope and actors	Authors involved in case study and related research project
1—REDD+ and tackling drivers of deforestation	Investigation of climate policy’s ability to tackle deforestation drivers and related profit, power, and accountability structures	Qualitative and quantitative analysis: Survey and interview data on discourses and policy networks at national level from 16 countries since 2009 Media analysis Policy document analysis Policy network analysis Qualitative comparative analysis (QCA)	Bolivia, Brazil, Burkina Faso, Cameroon, Democratic Republic of Congo, Guyana, Ethiopia, Indonesia, Laos, Mozambique, Myanmar, Nepal, Peru, Tanzania, Vietnam, and Papua New Guinea Global review, focus on large forest-rich REDD+ countries and driver structure in global North and South	Brockhaus, Di Gregorio, Moeliono, Pham, Wong Norway, EU, DFID, IKI/BMZ funded project: CIFOR’s GCS-global comparative study on REDD+ (GCS-REDD+) (2009–2020) https://www2.cifor.org/gcs
2—Development, Forestry, and Climate Policy	Examination of climate and social forestry policies in reflecting logics and discourses of development and forest and land governance control in the forest frontier	Qualitative analysis: Policy document analysis Analysis of interview and field survey data from 3 countries since 2010 (gender and age disaggregated), and from workshops at national and regional levels	Indonesia, Laos, and Vietnam Proponents of development, community forestry, conservation, and climate change policies representing global North, ASEAN, and national institutions	Wong, Moeliono, Brockhaus, Pham Swiss-funded project: ASEAN-Swiss Partnership on Social Forestry and Climate Change (ASFCC) (2010–2020) https://www2.cifor.org/asfcc/
3—Adaptation and a forest that no one wants	Analysing with an intersectional lens to understand the interaction of climate and development politics with vulnerability dynamics, adaptive capacity, and strategies of different social groups across multiple levels related to a novel forest ecosystem frontier	Qualitative analysis: Policy document analysis Intersectional analysis of focus group discussion and Interview data (gender, age, ethnic disaggregated) from 3 communities and at national and sub-national levels	Mali, Lake Faguibine Global, national, and local adaptation policy actors	Djoudi, Brockhaus EU-funded project: Tropical Forests and Climate Change AdaptationTROFCCA (2005–2009) https://www1.cifor.org/trofcca/home.html
4—Climate change adaptation, mitigation, and development	Analysing power in adaptation, mitigation, and development policy processes and local implications for forest frontiers across scales	Qualitative and quantitative analysis: Policy document analysis Analysis of interviews with climate change policy actors at three levels of governance Multi-level policy network analysis of survey data	Brazil, Indonesia Multi-level governance actors across national and two sub-national levels: in Brazil at national, state (Mato Grosso) and municipality level, and in Indonesia at national, provincial, (West Kalimantan) and district level	Di Gregorio, Brockhaus ESRC-funded project: Multi-level governance, REDD+ and synergies between climate change mitigation and adaptation (2013–2016) https://gtr.ukri.org/projects?ref=ES%2FK00879X%2F1

While not all authors collaborated across all research projects and sites (with the lead author as exception), most authors were affiliated to the same international forest research organisation, despite over different projects and periods of time. As authors, in our critical review of discourses, incentives, and power relations, we take an explicitly normative stance through the use of our political economy lens on human–nature relations in the Global South. This positioning draws attention to inequalities embedded in unbalanced power relations, recognises the political nature of socio-economic relationships, and puts ethical consideration centre-stage (Scoones et al. 2015; Klinsky et al. 2017; Clapp et al. 2018). Consequently, business as usual is defined as largely unsustainable and unjust, because it reinforces unbalanced power structures that favour large-scale business interests driving unsustainable practices and facilitating state capture (Rowley et al. 2013), while transformational change is specifically defined as a ‘just transition’ breaking up pre-existing power structures, reducing power imbalances, and empowering actors that support sustainability. This is not to say that there are no tensions between sustainability and justice, indeed part of the challenge of transformational change is to navigate ‘sustainability-equity’ tensions (Newell and Mulvaney 2013; Ciplet and Harrison 2020). Hence, our normative stance is reflected in conscious decisions over the choice of what and how we study climate governance and forest frontiers, and for whom. Finally, our long-term presence and collaboration in the selected sites enabled us to take a long-term perspective in the study of change over time.

As Table 1 shows, the cases differ in their specific sites, geographically as well as analytically, with Case (1): a forest mitigation case about REDD+ policy developments and voluntary commitments based mainly on national-level policy analysis across 13 tropical forest countries; Case (2): a development and climate change case drawing on local field data and policy reviews from Indonesia, Laos, and Vietnam; Case (3): an adaptation case anchored in local level research in the area of the Lake Fauibine in Mali, where a forest emerged after a lake system linked to the river Niger dried out. Here, the forest frontier is perhaps the least central to the actors involved in the case that investigates how herders and farmers have adapted over time in this silvo-agro-pastoral system; and Case (4) a case on the integration of adaptation, mitigation, and development efforts in two forest-rich tropical countries, Brazil and Indonesia. All studies are anchored within national policy but reveal important interactions with international and local representation and influences reflected in forest frontiers as discussed in the earlier section.

In each case, we apply the 4Is framework (Brockhaus and Angelsen 2012) and provide insights on how Institutions, Interests, Ideas, and Information as outlined below interact and enable or hamper transformational change:

- *Institutional* path dependence and stickiness limits change and is often linked to formal power structures (e.g. reflected in colonial land laws and rules, Ministries responsible for natural resources and extractive industries). Institutional change is necessary to break these structures in order to facilitate transformation.
- *Interests* refer primarily to economic and political interests. When state interests in social and economic welfare of society fall short it is often because of lack of autonomy from interests driving deforestation and degradation (e.g. reflected in profit and rent seeking, fraud, collusion, and corruption). Transformation usually requires a shift in incentive structures and power relations to ensure interests of some key actors change and serve societal needs and ambitions for just transitions.
- *Ideas*, including ideologies, worldviews, beliefs, and discourse, can reinforce the status quo, as they shape problematisations of environmental impacts and limit the set of choices of what is ‘reasonable’ or what is put forward as ‘the possible’ (e.g. benefits from forests for those who effectively and efficiently link local forests to global value chains, versus benefits for those who have moral rights based on equity considerations)
- *Information* is an important source of power, and data, knowledge, and evidence are often selected, interpreted, and put in context in ways that may reflect the interests of the information provider (e.g. when forest definitions are provided, land-use activities are monitored and rankings are established to distribute climate adaptation finance). Improved access to information or new information can contribute to shift power balances and facilitate change.

Building on our cases, we explore the ***institutional path dependencies and stickiness*** embedded in the rules, norms, and policies governing actors and action in the forest frontiers, paying particular attention to the continuation of colonial legacies in post-colonial states. Within this context, we then highlight actors’ diverse (material) ***interests*** and their ***ideas*** and ***information*** through an understanding of nature, resources, and commodities in the cases. Here, we pay particular attention to conflicts and collaboration, the role of knowledge and scientific advice as well as accessing and sharing of information and implications for accountability.

In all cases, we mapped the North–South or global dimension, and identified patterns that enable or hinder the larger changes needed to move away from inequalities embedded in business as usual resulting from the governance of forest frontiers, be it for adaptation, mitigation, or both and expressed in ongoing deforestation and maladaptation. Maladaptation here is defined as the result of an intentional adaptation policy or measure directly increasing vulnerability for either the targeted and/or for external actor(s) and/or eroding preconditions for sustainable development (Juhola et al. 2016). In each case, we explicitly asked what is missing in the way forest and climate change are problematised and how solutions are presented.

In the comparative and multi-level analysis, we then highlight common and distinctive features of the diverse dynamics of power and politics that became visible through our 4I lens, with issues ranging from expressions of agency and actions of resistance and compliance, specifications of benefits and burdens, to the diverse trajectories of change.

## Results: four cases on adaptation, mitigation, and development in forest frontiers over time: a 4I application

The four cases selected for the analysis are highly diverse, e.g. with regard to the particular climate change related policies at play, the specific frontier characteristics, and the processes of change taking place (or not). They also feature highly diverse actors and relations between these across different levels and scales. Another main difference between the cases examined here are the underlying assumptions that guided the analysis of the specific case material. Table 2 highlights these diversities and also provides an overview of the literature the original case study produced and on which we build our analysis here.

**Table 2:** An overview of the four cases highlighting the underlying research assumptions, describing the frontiers, the core element of transformational change analysed, and case-relevant key literature produced by authors

Case	Research assumptions	Frontier	Core element of TC analysed	Case-specific literature
1—REDD+ and tackling drivers of deforestation	Climate policies need to tackle deforestation drivers in the frontiers of the Global South that are linked to broader profit and power structures and political networks	REDD+ and other climate and forest governance policies aiming to reduce tree loss and degradation compete with political economy of drivers pushing deforestation in the frontiers in the tropics	Slow shifts in discursive practices, struggles over shifts in incentive structures, and sticky power relations: New knowledge on drivers challenges existing myths related to drivers of deforestation, new incentives available for standing forests, and information shifting power relations as triggers for transformational change Business as usual is often represented by state, and corporate and financial institutions are powerful actors as they control the capital invested in dramatically changing forest frontiers and are able to influence governments’ decisions and political cultures	Brockhaus et al. (2012a, b, 2013, 2014a, b, 2017) Di Gregorio et al. (2012, 2013, 2015a, b, 2017) Assembe-Mvondo et al. (2013) Korhonen-Kurki et al. (2014, 2019), Kaisa et al. (2017) Pham et al. (2019, 2021a,2021b) Gebara et al. (2020) Moeliono et al. (2020)
2—Development, Forestry, and Climate Policy	Climate change action risks to reinvent, maintain, or enhance existing ‘development’ interests and state control in the forest frontiers	Development, forest and climate policies are shaping peoples’ actions to fit political narratives and interests in the transitions of forest frontiers	Shifts in discursive practices and power relations (and to lesser extent incentive structures): Neither climate change nor social forestry policies are (yet) changing discursive practices. Social justice discourses are there but not yet dominant Resistance actions are present but not yet at scale (except Indonesia’s recognition of customary rights), rendered technical in bureaucratic tangles and projectification of indigenous claims Disproportionate incentives remain in place that motivate large drivers to deforest (concessions, tax incentives, and fast track policy decisions), and smallholders towards conservation (PES; REDD+)	Moeliono et al. (2017), Loft et al. (2017), Cole et al. (2017a, b, 2019), Pham et al. (2019), Kallio et al. (2019), Maharani et al. (2019), Bong et al. (2019), Thu et al. (2020), Wong et al. (2019, 2020), Sahide et al. (2020)
3—Adaptation and a forest that no one wants	Solutions to climate change adaptation are based on business as usual solutions, understandings, and politically convenient discourses of vulnerability dynamics often at the expense of strategies of different social groups in a novel forest ecosystem frontier in an arid region	Novel forest frontier creates new opportunities locally and provides a platform for a new commodity but as adaptation efforts unfold locally, it renders visible elite capture, colonial and post-colonial discourses, related interests, and hindering power structures	Shifts in discursive practices and local power relations, reinforced global power relations: Persistence of discourses of destruction related to pastoralism and mobility Solastalgia, longing for a forlorn past before extreme climate events (droughts), and the demise of the local institutions authority can represent a local barrier to change and adaptation, and can be exploited by politics in favour of business as usual Differentiated understanding of local needs call for shifts in climate finance architecture beyond shifts in incentives	Brockhaus and Djoudi (2008), Djoudi et al. (2013), Brockhaus et al. (2012a, 2013), Djoudi and Brockhaus (2011, 2016)
4—Climate change adaptation, mitigation, and development	Constellations of power across governance levels in adaptation, mitigation, and development policy processes differently shape equity and policy outcomes at the forest frontier	Impacts of global and national CC governance on climate and development in the forest frontier, and policy actors’ ability of delivering change/ climate compatible development s locally	Shifts in power relations through policy coalitions: Local climate change adaptation needs were not well understood, and demands for climate justice were mainly raised by the least powerful group of local development NGOs and remained neglected in policy practice impacting negatively marginalised and vulnerable groups Alliance needed between civil society and key reformist government actors willing to push climate change adaptation and provide a vision of an alternative and sustainable form of local development that caters to local needs and redirects attention of short-term interests of the national treasury and global markets to long-term resilience building in local communities	Di Gregorio et al. (2015a, b, 2016, 2017b, 2019), Locatelli et al. (2015)

One core narrative or **idea** persists across forest frontiers and efforts to halt deforestation and enable development: the suggestion that local actors, particularly smallholders and communities, as well as pastoralists, need to be turned into settled agricultural entrepreneurs to ‘develop’ and ‘modernize’ traditional grazing and shifting cultivation systems, which have been blamed as main drivers of deforestation (Dressler et al. 2017; Thu et al. 2020). The 4I analysis of each of the cases as summarised in Table 3, reflect this core narrative, and how it is mirrored in land-use **institutions** and the **interests** at play in the Global South (particularly in cases 2 and 3 on development and local adaptation to climate change). Despite evidence to the contrary (Ziegler et al. 2011; Dressler et al. 2017; Bruun et al. 2018), the framing of shifting cultivation and mobile husbandry, of pastoralists and peasants, as responsible for deforestation and degradation has persisted since colonial rule (Thu et al. 2020; Scoones 2021). Such ideas affect till today decisions over what counts as deforestation or does not, and they legitimise which drivers are defined as legal or illegal, measured and reported by whom, how databases are constructed and made accessible, and where are the blind spots—highly political questions as cases 1, 2, and 3 in particular show (see Supplementary information). In addition, as also case 2 highlights, these accounts are then presented as **information** to justify claims over forests for large-scale production of global commodities such as timber and large-scale agricultural commodities at the expense of local people, as other literature and evidence especially from Southeast Asia highlights (Dove 1983; Doolittle 2003; Dressler et al. 2017; Thu et al. 2020). The colonial discourse of unsustainability of the commons and the deliberate ignorance of customary or traditional farming practices is argued to be partially related to the government’s inability to collect revenues from these practices (Doolittle 2003) and is still today embedded in legal frameworks regarding forest and land tenure, as case study 2 with examples from Southeast Asia shows. Hence, one could argue that this **institutional path dependency** continues throughout the ‘post’-colonial state. The **interests** of former colonial powers and elites are hence firmly anchoured in legal frameworks of ‘resource governance’ (Assembe Mvondo et al. 2014; Dominguez and Luoma 2020; Astuti 2021). This interest representation leads then to a ‘global’ forest governance over tropical forests that seems to be more concerned with ‘Northern’ and capitalist interests representation and control. Demand for food, fuel, and fibre in the Global North, is served through states in alliances with large business interests (Kroeger 2016). Such powerful actors and alliances dominate the frontier dynamics, undermine local agency, and depoliticise forests by rendering the problem of deforestation a technical one (Li 2007; Peluso and Vandergeest 2020). Case 1 in particular highlights these processes, with analysis from Indonesia showing how forest-based mitigation was initially linked to large political change but is now reduced to a technical project (Moeliono et al. 2020), or in Brazil, where interests in keeping forest standing has been shifted over time towards restoration interests linked to intensified biomass production (Gebara et al. 2020). Simultaneously to these power relations of domination and oppression, visible in all cases presented in this paper, power struggles take place within localities, among farmers and herders struggling over access to resources and between state and citizens with diverse and conflicting visions of future development (cases 2 and 3). Power struggles also occur across levels of governance and interest (cases 1, 2, and 4), and across sectors and line ministries competing for limited land and financial resources (cases 3 and 4), including those provided through overseas development aid and climate funds for green growth, sustainable development, and mitigation and adaptation actions. Table 3 shows how the 4Is play out in our different cases, and provides incidents from the case study research and the wider literature, while more detailed descriptions of the cases can be found in the Supplementary information to this paper.

**Table 3:** Unpacking the selected cases: Institutions, Interests, Ideas, and Information (4Is) and incidents from case-specific analysis and wider literature (see Supplementary information)

Case	Institutions	Interests	Ideas	Information	Incidents from case study research
1—REDD+ and tackling drivers of deforestation	Colonial path dependencies in land rights, institutional stickiness of established business-as-usual institutions leading to contradicting objectives between climate and anti-deforestation policies with other extractive or development policies	Competing interests in forest frontiers, with state often representing selected versus societal interests, with financial incentives often in favour of business-as-usual interests	Discourses promoting specific modes of governance, with dominance of market-friendly and anti-regulatory approaches Persistence of colonial narratives in responsibilisation of smallholders as agents of deforestation New narratives on importance of divestments emerging	Information on drivers misleading as selected and constructed facts Information as a catalyst for changing power through transparency and consistency	Legitimisation of deforestation as an essential requirement for national economic development remains strong discourse across major forest biome countries (Brazil as most recent example, also supported in Pereira et al. 2019; Kroeger 2020) Revenue ‘sharing’ from forestry sector in Cameroon guided by relationships between state and private forest sector actors forged in colonial institutional legacies in favour of multinationals with European and Asian roots rather than serving the interests of affected local communities and the wider society Reviews of national REDD+ strategies and REDD+ practices in Indonesia, Vietnam, and globally indicate that deforestation is rendered a technical problem. Policy action disproportionately targets smallholders and is blind to large drivers of deforestation and forest degradation and the social injustices associated with these (Salvini et al. 2014; Myers et al. 2018; Skutsch and Turnhout 2020) State actors in Indonesia, Brazil, Vietnam, Cameroon, PNG, and Nepal often represent business-as-usual interests in media Commitments by state and private sector to halt deforestation are not substantiated by effective action, and incentives for deforestation in policy and practice continues in Brazil, Indonesia, Peru, Bolivia, Nepal, Cameroon, and Vietnam Investments in large land-use change continue, despite divestment efforts (Norway) Forest governance reforms in favour of local people remain limited (Indonesia, DRC, Brazil) In Indonesia and Brazil, deforestation in forested areas not gazetted/designated as forest is not counted as deforestation In ¾ of 17 countries, a review of national REDD+ policy documents shows that safeguards, gender-sensitive FPIC, and benefit-sharing mechanisms are lacking or still under development Policy networks in all investigated countries indicate that power typically rests with state actors lacking autonomy from established forest and land-use sector interests
2—Development, Forestry, and Climate Policy	Colonial path dependencies create/ maintain structures of territorialisation that legitimises large-scale deforestation as development in the forest margins at the expense of smallholder practices and customary rights	State and local people have a shared interest in ‘development’ but state-enabled development in forest frontiers have largely led to local dispossession and increased precarity with benefits flowing to external actors. Local interests in customary rights are largely ignored	Narratives reflecting colonial ideas of the deforesting shifting cultivators, and capitalist framings around particular ideas of development are mirrored in existing regulatory structures, and limited rights even in decentralised social forestry schemes	Narratives on political visions of capitalist development and economic growth are reinforced with biased information and are persistent over time, leaving little room for recognition of different knowledge systems, values of alternative development pathways	The states’ attempt to redress inequalities with policies of social forestry and PES/REDD+ incentives targeting smallholders and local populations in forest frontiers in all investigated countries, but old ideas and institutional pathways have largely rendered such ambitions technical: - social forestry in Indonesia has high bureaucratic requirements and the expectation of a new class of forest entrepreneurs that can generate profits for livelihoods, - social forestry in Indonesia comes with technical requirements that strengthens state control rather than a real devolution of rights, - REDD+ in Laos is ‘the same job with a different name’ aimed to stop shifting cultivation considered as the driver of deforestation, - PES in Vietnam is driven by administrative and hierarchical aspects of distributing incentives to responsibilise poor communities to maintain forests Language of social forestry and development is dominated by market references, with expectations that local people are capable entrepreneurs and conservationists with the right training
3—Adaptation and a forest that no one wants	Institutional path dependencies with regard to mobility in land use—sedentarisation policies, land reforms and formalisation of rights hindering shared traditional resource use practices	Locally diverging/ competing and favoured interests in transition from lake to forest frontier enabled by top down national policies shaped by global north development paradigms reflected in technofix solutions	Degradation/desertification narratives associated with pastoralism paradigms and vulnerability narratives influencing today’s land-use policies	Knowledge selected based on value statements with selected information determines adaptation narratives and praxis. Diverging perceptions on adaptive capacity and its determinant expressed in modernity myths versus place-based adaptation	Mobility, of people and their livestock conflicted with commodity needs and related ideas of an ‘agriculture rationelle’ assigned to colonial landscapes (e.g. cotton production in West Africa, *cotonialisme *(Roy 2012) and conflicts nowadays with post-colonial administrative boundaries, land-use rights, election cycles, tax years, and conceptualisations of what constitutes citizenship (Karambiri and Brockhaus 2019) Local actors portrayed mobility as an adaptive way of life to access various resources, while governmental representatives saw mobility as an impediment to development and adaptation Sedentarisation resonated with the modernity aspirations of young pastoralists as an attempt at emancipation from traditional systems Older generations of fishermen, farmers, and pastoralists alike longing for the long-lost rich lake system and expressed a sense of powerlessness or lack of control over the changes in the present (solastalgia) Dreams of a new future and a forlorn past are inspired by a techno-driven mega-project of ‘refilling the lake’ as solution to the experience of extreme outcomes of climate variability and change—being used and mentioned by politicians across levels as a promise, for electoral purposes, to mobilise funds for efforts to ‘bring back the lake’ Newly emerged forest resource (Prosopis) remains unmanaged and is used mainly by less privileged women Tensions in local aspirations bring out underlying assumptions and constraints to adapt to new conditions and climate variability in formal and informal institutions governing land and peoples’ relations
4—Climate change adaptation, mitigation, and development	Institutional path dependencies hamper cross-level policy processes that support local needs despite federal and decentralised regimes	Interplay between global, national, and local interests in shaping the forest frontier and resulting marginalisation of less powerful local interests	Dominance in discourses across scales, with global and national mitigation discourses dominating at the expense of local adaptation	Power asymmetries in information related to presence/absence of (and support of) capacity and access to information, with insufficient knowledge available on adaptation responses	In Brazil and Indonesia, knowledge gaps were much more extensive for adaptation than mitigation and were unequally distributed across governance levels, with expertise on climate change adaptation primarily located on a national level, when it was most needed at local level Lack of local expertise translated into the inability of local governments to effectively demand attention for and effectively address local adaptation needs related to droughts, floods, and fires in both forest frontiers, but particularly in Indonesia In both Brazil and Indonesia, climate change action is primarily funded through bilateral aid, from Norway, Germany, and the UK, while multilateral aid from the World Bank and United Nations agencies is also key in Indonesia In the state of Mato Grosso, forest governance featured powerful economic livestock and soy agribusiness interests supported by national-level allies and local landowners’ associations. Contraposed were long standing but less well-resourced environmental conservation and climate change mitigation interests. In West Kalimantan agribusiness-led development focusing on oil palm production was strongly supported by all government levels, relations with NGO were at times conflictual A local forestry agency in West Kalimantan adopted the climate change mitigation discourse to attract finance to deliver sustainable forest management and support local development. However, reducing carbon emissions was not its main goal

## Comparative analysis of change in the forest frontiers of the Global South

The cases provide deeper insights into the many ways of how the forest and climate change agenda is linked to unsustainable development, injustice, and inequality in the forest frontiers of the Global South. The cases are highly diverse in their geographies, spatial, temporal scales, levels of governance investigated, and the types of data analysed. Yet, politics and power are at play in all cases, and the common and differentiated features in the cases will allow us to provide some answers to our central question that guided this investigation. Particularly, we are interested in the understanding under which circumstances climate governance and related policies are able to facilitate transformational change away from a business as usual of inequality with ongoing deforestation and maladaptation.

### Unpacking politics and power within the cases

Case 1 on REDD+ puts into focus North–South relations and the agents and interests playing out in contested forest lands in the tropics. The case highlights how ignoring conflicts between societal and selected interests and the power imbalances present in the forest frontiers weakens efforts to halt deforestation. Private sector actors are often absent from formal REDD+ policy processes but their interests are vitally present, often represented by state actors (Di Gregorio et al. 2013, 2015a, b; Brockhaus et al. 2014b). Yet, while calls for the need to have the ‘private sector in the room’ are dominant, there are few policy actors calling for regulation of those private sector actors driving deforestation. Meanwhile, the voluntary policy initiatives, such as the New York Declaration of Forests, are a new playing field for these private sector actors (in commodity supply chains, trade, and investment) but fail to reach their own targets, have incoherent reporting (NYDF and Partners 2020) and, with little consequences and accountability, run the risk to distract from the core efforts of REDD+ and other mitigation efforts. It also seems that the politically palatable topic of engaging with the private sector and multi-stakeholder public–private fora has relegated earlier REDD+ ambitions for larger systemic change to small ‘projectified’ spaces. While power relations seem to remain in favour of business as usual, technological advances, and new information have provided alternatives to political reporting and outdated models of deforestation (e.g. those based on simple indicators of population density). The resulting new discursive practices in policy spaces concerned with deforestation seem to gain traction. In addition, increasingly more transparency along supply chains and within decision making can make it more difficult to defend the politics of deforestation.

Case 2 on development along forest frontiers in Southeast Asia highlights how post-colonial ideas behind the deforestation narratives and associated policies continue to reflect older discourses by responsibilising local communities to stop deforestation and forest degradation despite the significant role of large-scale deforestation drivers and the policies that enable them (Enrici and Hubacek 2016; Ingalls et al. 2018; Cochard et al. 2020). Together with limited devolution and lack of recognition of local rights, this points to state territorialisation with interest to maintain control over lucrative forest resources through a strengthening of the financial, political, and ideological control of remote populations, diverse cultures, and traditional land-use practices.

Case 3 on adaptation in agro-silvo-pastoral systems in West Africa introduces a forest that nobody wants while those using it face challenges related to institutions and discourses. The case highlights how mobility as a highly adaptive strategy is undermined. First ‘demonized’ in colonial discourses, the same rationales are used by post-colonial state elites to continue pushing for the sedentarisation of pastoralists. The herders and those who were once farmers and fisherman in the former lake region are hampered in adapting locally to the evolving forest ecosystem; narratives of reflooding the area are nurtured through promises of mega-projects’ techno-fixes and lead to no or maladaptation, with little or no institutional and financial support for optimising locally adapted strategies. Vulnerability, if not understood and framed in a socio-historical context and designed based on a deeper understanding of local complexities, will continue to result in and shape inequalities. As a tool in climate politics, it is then reduced to a simple means to attract funds for a constructed vulnerability, while, on the other hand, finance to support local adaptation remains missing. The powerful combination of discourses of modernity, longings, and aspirations combined with political and financial (self)-interests leads to maladaptive pathways. The case also shows how local actors seize new opportunities for adaptation and resist discourses and unsustainable promises based on beliefs in techno-fixes to climate change and extreme events.

Through the investigation of the linkages between forest-based mitigation, adaptation, and sustainable development processes, case 4 illustrates that power structures on the forest frontier in the Global South are in fact multi-level in nature. Local implementation is strongly impacted by global climate policy processes, rules, and norms, which has an inherent bias toward mitigation action. Further, the constellation of national and sub-national climate mitigation and adaptation interests, together with country specific federal (Brazil) and decentralised (Indonesia) institutional structures interact to shape local outcomes. Simultaneously, local actors use their limited resources, in particular discursive practices, to appropriate and reshape global and national practices. They articulate their interests, predominantly as developmental in nature, but climate change adaptation needs remain poorly addressed, because of a mix of lack of knowledge at lower government levels and lack of support from higher-level institutions. How can the three policy objectives of mitigation, adaptation and sustainable development be effectively integrated to deliver increased resilience to climate change and a sustainable form of development on the forest frontier? A rebalancing of power in policy processes is required to make them more inclusive of diverse local actors' needs and interests.

Common across the four cases, climate action has been often found to be absorbed and lost within development priorities of producing commodity crops for global markets, accompanied by techno-fixes, which are framed as expressions of modernity. Colonial hegemonies are perpetuated as climate action relies on institutional frameworks governing forest lands that newly created nation states have left unchanged with ‘independence’. Hence, these post-colonial states with their path dependencies provided fertile grounds for neo-colonialist framings of forest frontiers in the Global South. The adaptation case with the newly emerged forest exemplifies the continuity of ‘the colonial’ in forest frontiers, as its underlying discourses, institutional legacies, and financial dependencies are reflected even in a place that only recently was turned into a forest frontier.

All the cases pointed to policies that aim to halt deforestation driven by large-scale resource extraction and commodity production on forest frontiers and enable adaptation or enhanced adaptive capacities in the frontiers. Yet, across all cases, these efforts are countered by policies and incentive structures that continue to drive deforestation and maladaptation, often supported by the investments of powerful actors. We also learned about the new commodity of a constructed vulnerability, in contrast to the many lived forms of vulnerability characterised by loss of assets and lack of support to access new ones. Here, vulnerability as commodity, while intangible and constructed by diverse interests often disconnected from the local level, serves national elites in their negotiations over climate finance. Meanwhile, root causes of vulnerability remain unchallenged and an ‘Olympics of Vulnerability’ continues with rankings and indexes to determine those most vulnerable (Djoudi et al. 2016; Djoudi and Brockhaus 2016; Barnett 2020). The dominance of business-as-usual trajectories of large-scale deforestation and maladaptation is overwhelming in all cases. Transformational change from these patterns seems out of reach, despite the many well-intended policies and measures that are part of forest and climate change governance in these forest frontiers.

### Ways forward to realise just transitions: Or powerful echoes from the past favouring selected interests?

Across the cases, business as usual in favour of selected interests has maintained dominance over time. While this might not be surprising, the 4I lens we applied to the cases focusses potential leverage points for change in the interplay of diverse factors in the production and reproduction of inequality, in the sharing of benefits and burdens from the changing forest frontiers, and regarding the urgent needs for climate action.

All four cases emphasised how colonial and post-colonial institutional path dependencies enable the continuation of established North–South power relations, with national elites reinforcing these to pursue their own political and economic interests. Discourse shapes these institutions and the related policies and practices as they provide justification for selective priorities and preferences, for e.g. when mitigation matters more than adaptation or when cash crops for global needs are prioritised over local adaptive livelihood and mobility strategies, often summarily dismissed as backwards and environmentally destructive. These findings corroborate much of the literature on political ecology as well as political economy (Dauvergne 1993; Peluso and Lund 2001; Rudel 2007; Forsyth and Walker 2008; McCarthy and Tacconi 2011). In the adaptation case study, we characterise one of the processes that enables this as the set-up of an echo chamber, where the power of a discourse is built on a complex machinery of repeated myths through government speech and officials educated by the very interests that benefit from the practice that follows the myth and echoed by many people whose dreams and ambitions resonate with what is or was promised by the myth. Science and scientists risk to be part of such echo chambers, too, when landscapes are constructed on land-use practices and measurements based on Western forestry ideals and experiences, and expressed through formulas of plantation productivity. Then, science might mute social justice concerns and overpower diverse local land governance practices and voices that call for a critical examination of whose interests are being served by forest management practices, policies, and measures (Ribot et al. 2006; Peluso and Vandergeest 2020). The power of these echo chambers becomes even more audible when local farmers and herders describe their own practices as environmentally harmful, sometimes in self-deprecation and other times as a belief.

The cases also highlight what is not problematised and what is missing in framings of climate action along forest frontiers. Across all four cases, the local and the political disappears over time and forest and climate governance in forest frontiers is rendered technical (Myers et al. 2018; Skutsch and Turnhout 2020) and reduced to capacity issues. Instead of engagement with local practices, diverse knowledges, and needs, there is a call for administrative, technical, and financial assistance from the international community. The forest frontier then becomes a depoliticised yet re-colonised site of established North–South relations that date to colonial times in both practices and mindsets.

Power is central to all these processes and to argue, following Foucault (1978), where there is power there is also resistance. ‘Power over’—coercive authority—is visible throughout the frontiers presented in our cases, often in favour of selected interests and in ignorance of local realities and needs. Yet, we do also see ‘power to’—the productive and generative side of agency—expressed across our cases. The REDD+ case is an example where this constant struggle between interests, coercion, and resistance takes place. Here, we see how, after some initial impulses and calls for broader change, over time climate change actions are seemingly retrofitted into the existing global neoliberal economic order and no longer contribute to nor aim to transformational change. Nevertheless, there is growing acknowledgment that this very order is the source of the problem (Delabre et al. 2020): a barrier to action, a threat to agency’s ‘power to’ change, as we saw in the adaptation and development cases; and a root cause of the problem in the case of REDD+ with global trade and investment patterns driving deforestation beyond tipping points (Galaz et al. 2018). In the adaptation case, an outlier in our initial framing of forest frontiers and related extraction and production of resources and commodities, we argue that vulnerability is also fitted to be part of the current economic order but also reflect constant struggles between the powerful and the powerless. The case demonstrates how a North–South dynamic plays out indirectly in a newly emerged forest, with state actors being occupied by efforts to gain access to and oversight over promised global climate adaptation finance by contributing to the construction of vulnerability as a commodity, yet, while this might indicate a strategy of the post-colonial state to play the existing system, this takes place at the expense of a focus on unpacking local needs and strategies in light of already scarce climate adaptation funding. This struggle and the related trade-offs were also evident in both the REDD+ and the integration cases. In summary, our comparison of different climate policies and interventions across the Global South shows the challenges any type of intervention in forest frontiers faces, and the risk that climate policies actually deny diversity and self-determination at local levels, while also reducing autonomy of post-colonial states from selected global interests in forest frontiers. While we saw differences in the outcomes of these interventions, the overall tendency is that the forest frontiers continue to reproduce inequality, loss of forest, particularly old-growth forests, and maladaptation to the disadvantage of those directly living in or depending on forests—in spite of, and in part because of, climate policy interventions. These findings are corroborated by observations in the wider literature on environmental and climate policy and its outcomes (Dawson et al. 2018; Martin et al. 2020), which also led to questioning the overall contemporary framing of environmental policy and science with its lack of attention to justice, democracy, and inequality (Biermann 2021). What is missing in all cases is a prioritised and powerful interest in keeping trees and forest standing, to the benefit of local populations as an explicit part of just transitions with the aim to reduce existing inequalities, rather than as an afterthought or a desirable side-effect as part of complex—and often dangerous—net calculations (Delabre 2020; Carton et al. 2020).

The comparative analysis presented here is limited as it builds on existing data, rather than as an explicit research design. In addition, this attempt to bring together 20 years of research across the Global South and diverse policy arenas in one research paper also means that we lose some of the deeper insights and nuances from the individual cases. Nevertheless, in applying a wider political economy framework to the analysis, centred on institutional path dependency, interests, ideas, and information, the cases contribute to a better understanding of the processes that enable production and reproduction of inequality within and between South and North, when these forest frontiers are transformed and governed as resource and commodity frontiers.

## Conclusion

Across the forest frontiers presented here, initial calls for transformation and restructuring of trade, and of states for effective, efficient, and equitable climate policies are not sufficiently acknowledged and at times have been silenced. We have argued that transformational change requires substantive shifts in incentive structures, discursive practices, and power relations. The cases highlight initial attempts to change incentive structures to keep trees and forests standing and to break with existing myths and discursive practices. However, governments are dependent on state revenues to actively steer their own economies, and whether economic development will be sustainable, climate friendly and equitable may depend on their ability to resist the interests that are benefiting from the status quo. Only governments that are open and responsive to civil society and their citizens, are able to gain autonomy from large-scale economic national and international interests, and—allied with other reformist policy actors—might be able to initiate such change. Most often, such alliances require a vibrant civil society, driving, leading, and pushing for change, and being able to hold accountable decision makers to their promises. Thus far, these coalitions for change are not yet sufficiently powerful nor vocal enough to overcome BAU and its echo chambers (Brockhaus et al. 2014a, b). Fall-backs into comfort zones of established (and profitable) power relations put at risk efforts for any lasting change (Barr et al. 2010; Moeliono et al. 2020).

Power structures and institutional environments require nuanced analysis as they cannot be dichotomously organised in ‘good’ or ‘bad’ categories. While acknowledging this, the paper aimed to highlight in broad features what seems to serve selected versus societal interests. It also explored what supports today's unsustainable business as usual with ever-increasing emissions and inequalities and pathways that might be transformed for desired changes. As power structures and institutional environments are sticky but also dynamic, established powers are constantly challenged by new ones, and new coalitions and alliances take shape over time, new opportunities and openings arise for transformational change, both through and towards, explicitly equitable as well as effective climate policies. The institutions of our forest frontiers evolve constantly through different historical moments and periods. While the past itself cannot solely be blamed for today’s climate inaction, its manifestations over time do not yet allow actors to seize opportunities leading to major institutional change in these forest frontiers. It seems that where the colonial, the post-colonial, the legacies, and the newly created neoliberal institutions have built on each others’ power structures to realise their common interests, there is little working in favour of effective and equitable climate action. Colonial models of commerce and governing companies still vibrate through today's vision of what forests are, whom they should serve, and who is considered a risk to efforts that aim to realise these visions. Climate governance in the four forest frontiers presented here, with ambitions to deliver mitigation, adaptation, and development, has not been able to break with established discursive, incentive, and power structures.

Nonetheless, our cases also highlight that climate change policies and measures can contribute to desired changes: with shifts in discursive practices and the incorporation of new or different forms of knowledge; or with shifts in incentives and power structures, e.g. facilitated by increased transparency and accountability; or with policy action that removes incentives and benefits for those driving large-scale deforestations. They can contribute to shift the dynamics of current forest frontiers characterised by resource exploitation and commodity production. There are alternative representations or framings of the deforestation problem and growing diversity of voices at different governance levels that question the divisions of benefits and burdens related to forests. The clamour of alternative discourses and interests across different actors and sectors is a welcome challenge to the dominant techno-scientific and political echo chambers. Yet, while in search of responses to the wicked problems of deforestation and maladaptation in the Global South, forest and climate policies have to address the underlying problem and causes of deforestation and maladaptation rooted in unequal power relations, supported by dominant and persistent narratives and expressed in unjust distributions of benefits and burdens from tropical forests and climate change action. Failing to challenge the wider political and economic system governing forest frontiers and focussing only on symptoms and isolated solutions, as the examined forest and climate cases show, climate policies risk to maintain and produce social and environmental injustices that characterises business-as-usual in forest frontiers in the Global South.

## Supplementary Information

Below is the link to the electronic supplementary material.

Supplementary file1 (PDF 780 kb)
