# Supplementary material for: The forest frontier in the Global South: Climate change policies and the promise of development and equity
Source: Ambio. 2021 Sep 3;50(12):2238–55. doi: 10.1007/s13280-021-01602-1 (PMC8563894; doi:10.1007/s13280-021-01602-1)
Supplement: Supplementary file 1 — Supplementary file1 (PDF 780 kb) [file 13280_2021_1602_MOESM1_ESM.pdf]

Ambio

Electronic Supplementary Material: **Cases 1-4**

Title: **The forest frontier in the Global South: Climate change policies and the promise of development and equity**

Authors: Maria Brockhaus, Monica Di Gregorio, Houria Djoudi, Moira Moeliono, Thuy Thu Pham, Grace Y. Wong

Case 1. Mitigating climate change through avoided deforestation in the South: The challenge of tackling large-scale drivers

Since the early 2000s, the idea of incentivising tropical, forest-rich countries to keep their trees standing to help reduce emissions from avoided deforestation and other land use activities (REDD+) has been a firm part of the global climate change agenda, together with other initiatives such as the New York Declaration on Forests aiming at zero-deforestation supply chains (Seymour and Bush 2016).

This case examines how ***institutions and ideas over forest frontiers in the Global South are shaped by diverse agents over time*** and how this might legitimise deforestation, rather than contribute to promised objectives of keeping trees and forests standing**.** Historically, deforestation in the tropics has been largely driven by resource extraction of timber or minerals, as well as commodity production, for global markets often through colonial relations and **institutions**. These institutions also pushed for establishment of forest plantations with 'commercially interesting' species such as teak, accompanied by discourses promoting this model and the **idea** of scientific forestry as 'progress' (Bryant 1996; Galudra 2009; Vandergeest and Peluso 2006). By the 1970s, tropical deforestation had further accelerated mainly due to forest land conversion for commodity production such as soy, oil palm and beef (Geist and Lambin 2002; Pendrill et al. 2019). Governments also encouraged deforestation through policy and practice, legitimising deforestation and land claims as an essential requirement for national economic development; Brazil is a recent example (Pereira et al. 2019; Kroeger 2020). Colonial accounts and early assessments of global deforestation by the Food and Agricultural Organisation (FAO) presented rural populations and in particular shifting cultivators as the main drivers of 'environmental destruction', with population densities serving as a proxy for deforestation rates in FAO models (Rudel and Roper 1996). A growing body of more accurate estimates of forest loss (Curtis et al. 2018) and analysis of direct and indirect drivers (Angelsen and Kaimowitz 1999; Kaimowitz and Angelsen 1998; Meyfroidt et al. 2013; Geist and Lambin 2002) informed initial REDD+ plans and the underlying idea in RED(D+) to provide (through a carbon market system) an alternative stream of revenue for national treasuries in forest-rich countries and inform decisions of whether or not to award forest concessions for

extraction, plantation forestry or land use change for commodity crops (e.g., to oil palm). Yet, current practices in REDD+ have largely failed to tackle large-scale drivers of deforestation (Di Gregorio et al. 2012; Salvini et al. 2014, Moeliono et al. 2020).

Central features in the **institutional** and policy environment found in most REDD+ countries are the many contradictions (and budgetary imbalances) between policies supposedly enabling action to halt deforestation and those providing perverse incentives for deforestation-driving activities (Di Gregorio et al. 2012). Over the past decades, the push for globalised, market-based and voluntary approaches by public-private partnerships or private actors has also reached the forest sector (Pattberg 2005; Grabs et al. 2020; Hrabanski 2017), supported by narratives of government failure and a problematization of regulatory policies as the cause of destructive logging and ineffective sector operations (Duncan 1994; Dauvergne 1993). In fact, part of the early success of the REDD+ idea (Stern 2007) was the expectation that it could be implemented through market incentives and while there is some emphasis on policies and measures, it represented a move away from a traditional command and control approach. The most common voluntary instruments currently present in many tropical forest countries include roundtables to improve industry standards, certification and zero-deforestation commitments (Pacheco et al. 2018; Pirard et al. 2015; May et al. 2016; Pham et al. 2019; Benn et al. 2020; Thaung et al. 2020; Dkamela et al. 2010; Mpoyi et al. 2013; May et al. 2016; Babon et al. 2013). Yet, recent evaluations suggest that documentation of concrete implementation and achievements with regards to avoided deforestation still falls far short of their own targets (Climate Focus 2016, NYDF Assessment Partners 2020). Reliance on market forces to control deforestation activities were also found to undermine agency of affected local communities (Larsen et al. 2018) to hold private and state actors accountable to their commitments. The over-focus on voluntary initiatives while undermining public regulations in high deforestation risk areas have potentially facilitated powerful actors to continue clearing forests (Pasiecznik et al. 2017; Lambin and Thorkalson 2018; Garrett et al. 2019).

At the same time, ***actors' interests and information*** are partly grounded in the conceptualisation of a 'sovereign nation state'. The early framing of REDD+ as a global Payments for Environmental Services (PES)

program operating at the national scale, might have by design led to less attention on the role of private actors driving deforestation and the global nature of multinationals and commodity value chains. This can become problematic in the logic of hybrid and voluntary governance, which is often dominated by vested **interests** and the power inherent in flows of investments and commodities (Kramarz and Park 2016). Despite advances in our understandings of land use teleconnections, spillovers and increased supply chain monitoring (e.g. with TRASE), transparency and accountability are likely to lose their potency to trigger larger change, as what is made transparent to whom might serve benchmarking or simple self-monitoring interests (Meyfroidt et al. 2020; Mol 2014; Gupta et al. 2020). Further, when defined behind closed doors, what is being accounted for is likely to serve only the goals of those who are invited (Kramarz and Park 2016; Mason 2020; Pasiecznik et al. 2017). In our case research, this 'shadow-network' of private sector actors currently driving deforestation became apparent as they were considered by other policy actors as important nodes in the REDD+ policy networks - yet they themselves did not share this perception or simply refused to participate in any research. We observed this 'absence' in REDD+ policy, practice and research across all countries, mainly of actors representing large drivers of deforestation, such as oil palm, soy and beef, timber and pulp and paper companies in Indonesia, Brazil and the Congo Basin (Brockhaus and Di Gregorio 2014, 2015a; Brockhaus et al. 2014; Moeliono et al. 2013; Dkamela et al. 2014). Largely absent from REDD+ debates at domestic levels are also those whose capital is invested in frontier transformation and who benefit from deforestation in the tropics without being necessarily fully aware of this (Cuypers et al. 2013; Di Gregorio et al. 2012; 2015a; 2017a; Brockhaus et al. 2014; Galaz et al. 2018). The Norwegian pension fund was an example of the disconnect in how the fund invests in and gains profits from a portfolio of deforestation-driving activities, while at the same time the country is also actively investing in halting deforestation, both ideational and financial. The Norwegian pension fund now leads other funds in pursuing divestment strategies, combined with active fund management.

These networks of financial, material and ideational flows are often rooted in colonial legacies of legal frameworks that led to state claims of forest lands, and the move away from collective ownership towards selected private actors (Rudel 2007; Logo et al. 2020), as seen in Indonesia (Peluso and Vandergeest 2020)

and more recently in Bolsanaro's Brazil (Pereira et al. 2019; Kroeger 2020). A project case study from Cameroon that analysed revenue sharing from the forestry sector shows how a combination of colonial institutional legacies and the relationships between state and private forest sector actors including multi-nationals with European and Asian roots has undermined 'benefit' sharing and, rather than serving the interests of affected local communities and the wider society, they mainly serve selected interests of the few (Assembe-Mvondo et al. 2013).

In the practice of REDD+ project implementation, major drivers of deforestation as targets for action have also become less visible, and **information** on these drivers is either ignored or underplayed, as we see REDD+ proponents across the world tackling the problems related to deforestation as mere technical challenges (e.g., getting MRV in place, designing procedures of distributing benefits) (De Sy et al. 2018). In reviews of national REDD+ strategies and REDD+ practices, deforestation appears to be rendered as a technical problem targeting smallholders, and agents seem to be blind to major drivers of deforestation and forest degradation and the social injustices associated with these (Di Gregorio et al. 2013; Salvini et al. 2014; Myers et al. 2018; Moeliono et al. 2020; Skutsch and Turnhout 2020).

From business as usual to transformational change

In earlier accounts of the REDD+ evolution, we noticed new coalitions of actors calling and working for action to halt deforestation. Many of these also came together while sharing concerns over equity, effectiveness, and efficiency, e.g. related to violation of indigenous peoples' rights, offsetting, 'paying the bad guys', or worries that REDD+ would take away attention (and funding) from long-established forest sector activities. Simultaneously, technical advances also moved a more radical change agenda, as they allowed a move beyond 'political reporting' and myths of who and what drives forest loss. Yet, this initial honeymoon phase was quickly over in countries such as Brazil, Indonesia and elsewhere when policies and measures tackling major drivers were supposed to be implemented. While power to change the current business-as-usual of deforestation lies primarily with the nation states and societies, in South and North, it is important to acknowledge that they face corporate and financial institutions that might be the most powerful stakeholders

as they control the capital invested in dramatically changing forest frontiers and influencing governments' decisions and political cultures.

Case 2. Forest, climate change and development: "Old" ideas in "new" policies in Southeast Asia

This case examines a forest frontier with a legacy of interests in resource and commodity extraction in Southeast Asia. Here, climate policies are very much framed in relation to existing policies of development and forestry, with climate change mitigation focussed on reducing deforestation. Much of the remaining forest in this region is located in areas where local people's livelihoods have long been subject to conservation, rural development and land conversion policies for commodity production and infrastructure development. Climate change mitigation policies are piled upon existing ambitions, expectations and policy objectives for these landscapes (Brockhaus et al. 2012). In the forest frontier areas, smallholders practise a mix of subsistence and commercial agriculture involving shifting cultivation, small-scale commodity cropping and customary forest governance practices (Heinimann et al. 2017). Southeast Asian governments typically consider these areas as backwards and in need of development, and have designed and implemented forest and land use policies that encourage only a form of development that prioritizes productivity, market access and entrepreneurship (Mertz et al. 2009). This discourse is reflected in the Association of Southeast Asian Nations (ASEAN) Economic Community (AEC) policy implemented in 2016 that aims to achieve a single market and production base for the free flow of goods, services, investment, capital and skilled labour within countries in the ASEAN. The underlying idea for the AEC is to improve the region's competitiveness as part of the global value chain (Cole et al. 2017b). Hence, we ask how Southeast Asian ideas of development interplay with climate change and forest objectives in Indonesia, Laos and Vietnam and what this means for the interests of actors and institutions involved. In all three countries, we find similar objectives underlying key policies around forests and forest lands, that is to enable a market- and (green) growth-driven economy.

Our reviews of policy documents find that the deforestation problem is represented by poverty, unproductive land/forest use practices and lack of formal land tenure and we discuss the implications of such problem representations for the forest frontier. Furthermore, we ask how this might create limitations but also provide opportunities for the role of climate change policies.

The legacy of colonial interests and ideas of productive land use

These representations reproduce colonial framings or **ideas** of the deforestation problem that has existed for centuries. The colonial narrative portrayed local smallholders and communities as responsible for deforestation because of their shifting cultivation practices (Dove 1983; Thu et al. 2020). These discourses are based on the contention that other uses of the forests, for timber or cleared for plantations, are less wasteful only because they yield taxable revenues for the colonial state (Doolittle 2003; Fox et al. 2009). Despite evidence on the sustainability of long-fallow shifting cultivation and its contribution to resilient livelihoods (Ziegler et al. 2011; Dressler et al. 2017; Bruun et al. 2018), the same narratives continue to prevail in contemporary political discourses, policy strategies and project practices around forests and climate change mitigation (Weatherley-Singh and Gupta 2015; Skutsch and Turnhout 2020). We examine how these discourses are reproduced in Indonesia, Laos and Vietnam and consider whose interests are served in forest and climate change policy solutions.

In Indonesia, social forestry is the basis for climate change mitigation and is accompanied by a discourse of "building the nation from the margins" with the underlying **idea** that providing access to land, business opportunities and vocational training will lift poor rural farmers and forest communities from poverty (MoEF 2018) and hence also help to maintain forest. The social forestry policy has been introduced and promoted by international organisations since the 1980s and is now supported by civil society and customary rights groups who have long advocated for the devolution of rights. Despite their engagement, research findings suggest that the **institutional** design of social forestry programmes with its restrictive land uses and limited rights serve only to reinforce the State's **interest** and control over forest land and transfer the responsibilities of forest management and conservation to local communities. This comes with the

expectation that communities develop forest-based enterprises to sustain livelihoods (Moeliono et al. 2017; Royer et al. 2018; Bong et al. 2019).

Laos' set of policies on forest zoning, resettlement, agricultural intensification and market integration implemented in the 1980s was considered a strategic solution to address the threat of a downward spiral between poverty and environmental degradation and "rationalise" land uses as a way of stopping shifting cultivation (Lestrelin et al. 2012). These policies fitted within global **ideas** of sustainable development and conservation and received substantive international aid and technical support. Updated versions of these policies have been harnessed for the commodification of land and resources under the Turning Land into Capital program (2006-2016) (Kenney-Lazar et al. 2018a) with the idea that market-based economic growth is the way to achieve the country's long-term **interest** of graduating from least developed country status while also reducing the practice of shifting cultivation in upland communities (Cole 2017a). In research of Laos' changing upland landscapes, we find that integration of smallholder farmers into market systems has generated high expectations of income that are often not met, rather such policies have often led to loss of farmlands and increased vulnerability of smallholders without the agency to fully participate in and benefit from these systems (Cole et al. 2019; Kallio et al. 2019; Nanhthavong et al. 2020).

The Payment for Forest Environmental Services (PFES) policy in Vietnam grew out of projects financed by international development programmes with market-based approaches for stopping environmental degradation. Initially resistant, the socialist state has adopted a narrative of success (To and Dressler 2019) promoting the **idea** that economic incentives to forest owners will motivate protection of forests, generate a stream of revenues to the Forest Department and support poverty reduction goals despite the fact that "ownership" refers to limited use rights and forest clearing or shifting cultivation in these areas are prohibited. As the incentives often do not sufficiently cover the costs of forest protection, nor livelihood needs, the narrative also relies on socialist framings of collective responsibility and an entrepreneurship approach to advance the State's objectives and **interests** (Vietnam Forestry Development Strategy, 2006-2020). In the process, technical, hierarchical and administrative aspects of distributing PFES benefits have

largely served the national narrative and ignored local ideas of equity (Pham et al. 2014; Loft et al. 2017; To and Dressler 2019).

When old ideas meet new institutions and information

Vestiges of colonial legacies and pervasive narratives of communities in frontier regions as being unproductive and under-developed have been adopted by the current governments of Indonesia, Laos and Vietnam; furthermore, they are reinforced through the incentives and **information** of climate change mitigation policies. Indonesia, Laos and Vietnam have embraced politically friendly "green-" and "low-carbon growth" development models with climate mitigation efforts and REDD+ largely pushed on to their existing social forestry, shifting cultivation stabilization and PFES agendas respectively (Dwisatrio et al. 2021; Indrarto et al. 2012; Lestrelin et al. 2013; Pham et al. 2019). In doing so, the state has further **institutionalized** the same narratives that prioritize national economic interests. The AEC and its aim to increase regional competitiveness will likely tighten the pressure on forests and smallholder farmers in forest frontiers, with expanding plantations and intensive practices to produce for the region potentially generating some economic opportunities and mobility while also increasing vulnerabilities. Thus, smallholders' precarious circumstances will be exacerbated as they are further marginalised (Cole et al. 2017b).

From BAU to TC

Local communities are however increasingly expressing agency in their demands for equity and rights as part of a more radical transformational change agenda in the forest frontier; their efforts have gained attention and at times led to reforms that were reluctantly instituted as in Indonesia's recognition of customary rights (Rachman and Siscawati 2016; Myers et al. 2017). Further, the diverse reactions, negotiations, contestations and resistances observed at various levels (Turner and Caouette 2009; Kerkvliet 2014; Kenney-Lazar et al. 2018b; Baird 2017; Setyowati 2020) and collaborations with civil society may be potential levers for transformational change of development discourses. They may even re-orient the lens of development planning toward local concerns. Whether sustained, political action of these levers can enable meaningful

and inclusive participation in the implementation and processes of climate change policies and drive the desired trajectory of rural change remains to be seen. However, without them, the old development discourses will surely continue to dominate.

Case 3. The forest that nobody wants: adaptation and maladaptation at a forest frontier in Northern Mali

The forest frontier in Northern Mali that was investigated in this case study differs from the other cases as it is situated in the West African Drylands, around a "created" forest initially planted around the former lake area as an anti-desertification measure to adapt to climate change in the 1970s. As part of our investigation, we focused on the local resources that were gained and lost with the emergence of forest. We used the 4I lens to highlight how, in a much more global setting, intangible resources are created and narratives are played out in this somewhat mystical forest in the region of Tombouctou (Djoudi et al. 2013).

The alignment of colonial legacy, scholarly paradigms and forest landscapes

Forests and trees play an essential role in adaptation to climate change in the Global South, by providing different benefits to the most vulnerable (Pramova et al. 2012; Koffi et al. 2017). In the drylands, this connection between forests and adaptation evolved through incessant adaptive learning in response to high climate variability, which is characteristic of the agro-sylvo-pastoral environments. The mobile nature of livestock herding is a vital element in the high adaptive capacity of those systems yet it features prominently in often one-sided desertification and degradation narratives that have dominated drylands discourses for decades (Reenberg 2012; Toulmin and Brock 2016). Mobility involves large spatial scales, multi-level institutions, diverse interests and the resulting collaborations (Turner 2010; Nori and Turner 2019). Furthermore, conflicts must be constantly negotiated through fine-tuned agreements, evolving values, rules and knowledge systems (Niamir-Fuller 1999; Nunow 2000).

In colonial times, the mobility of people and their livestock in large and ever-changing spaces conflicted with commodity needs assigned to colonial landscapes (e.g. cotton production in West Africa); more recently, it conflicts with post-colonial administrative boundaries, land use rights, election cycles, tax years and conceptualisations of what constitutes citizenship (Turner 2011, 2017; Painter et al. 1995; Karambiri and Brockhaus 2019). Physical landscapes that have been fragmented by the introduction of specialised commodity production with irrigation-based agriculture and protected areas, are also eroding and fragmenting the herding systems' **institutions** that represent the very fabric of a highly adaptive resource management. Over time, colonial laws and post-colonial policies have pushed for sedentarisation, and reduced pastoralists' access to traditional resources; this has been justified with discourses about livestock and mobility as a threat to development and the sustainability of natural resources and challenges the pastoralist way of life and practices (Heyd and Brooks 2009; Gonin and Gautier 2015). The **idea** of a "dangerous" pastoralism finds its continuity later in the desertification and degradation paradigms and the subsequent afforestation agenda that has dominated sustainability science in recent decades (Turner 2017; Scoones 2021).

Consequently, while there is an expectation to facilitate local adaptation through global climate governance (e.g., within the UNFCCC, National Adaptation Plans, the Adaptation Fund and other funding instruments), the often conflicting institutions and discourses at play in the adaptation arena (Mikulewicz, 2020) both hamper adaptation efforts and create instead maladaptive pathways. Maladaptation here is defined as the result of an intentional adaptation policy or measure directly increasing vulnerability for either the targeted and/or for external actor(s) and/or eroding preconditions for sustainable development (Juhola et al. 2016).

How ideas and promises enable and hinder adaptation

A contextual understanding is crucial when investigating the evolution of these tensions and conflicts between adaptation and maladaptation in the Lake Faguibine area in Northern Mali (Brockhaus and Djoudi 2008; Brockhaus et al. 2012, 2013; Djoudi et al. 2013). Lake Faguibine is part of a Niger River-fed lake system and was once a productive area for farmers, fishers and pastoralists; it served as a '*grenier du Mali*', providing

the country with food. The lake has been almost completely drained since the mid-1970s (UNEP 2009). Prosopis, a drought-resistant tree species, was initially introduced against desertification and has occupied the former lake (Brockhaus and Djoudi 2008; Laxen 2007). Ever since, several programmes have sought to 'bring the water back' and restore water-based economic activities (UNEP 2009).

Within the region surrounding this former lake area, many of the above-described historic tensions over land use and practices are reflected and shaped by different understandings of vulnerability and adaptation (Djoudi and Brockhaus 2011; Djoudi et al. 2016). Strong differences occurred in the ways different actors with diverse **interests** have framed vulnerability based on a wide range of implicit and explicit assumptions and **information**. These constructs then formed part of the wider justification for responses and adaptive strategies. Local informants portrayed mobility as an adaptive way of life to access the diverse locally and regionally available natural resources, while governmental representatives saw mobility as an impediment to development and adaptation. This illustrates how deeply local and indigenous systems have been discredited and what was initially instrumentalized to implement colonial and postcolonial control over indigenous territories has now become a firm element in state knowledge of vulnerability and its root causes. This was consistent with the aspirations and interests in the intensification of agriculture including livestock and the move from locally adapted subsistence agriculture to intensified practices has in fact enabled privileged actors to gain more power and access more resources. In workshops to discuss climate adaptation in the different communities, especially the younger and older men brought forward how sedentarisation resonated with the modernity aspirations of young pastoralists as an attempt at emancipation from traditional systems. However, we also learned how older generations of fishermen, farmers and pastoralists alike are experiencing *Solastalgia* (Albrecht et al. 2007; Tschakert and Tutu 2010), the longing for a distant past of a rich lake system. These were characterised by a sense of powerlessness or lack of control over the changes in the present. Dreams of a new future and a forlorn past are inspired in the narrative of reflooding the lake system as a technological solution to the experience of extreme outcomes of climate variability and change. The idea of "refilling the lake" resonated in the imaginaries of different actors in diverse ways but they all reflected strong beliefs in the promise of finding prosperity in modernity through technological fixes.

As researchers, we wondered how the idea of refilling the lake came about and while we were unable to determine the origins of the idea, we find that despite all the many unknowns regarding the causes of the drying up of Lake Faguibine, national and development actors are engaged actively in perpetuating the idea that this intervention is technically possible and by "opening" channels between the Lake to the Niger river, the past prosperity for fishermen and farmers will return. The wish for a 'return of the lake' has been used and mentioned by many politicians as a promise, for electoral purposes, and to mobilise funds to 'bring back the lake'. The employment of this discourse by powerful actors also fuel maladaptive pathways on a larger scale, as the sustainability of the lake's recovery is uncertain with ongoing climate change and recurring extreme climatic events such as droughts, and lack of attention (and incentives) for long-term strategic planning around the now available forest resources. Therefore, the discourses contribute to a politically constructed vulnerability with both local people and actors at meso and macro levels acting as echo chambers of techno-beliefs and mal-informed and hence maladapted Western management practices. Furthermore, rather than addressing the root causes of vulnerabilities and tackling existing inequities, vulnerability has been turned into a bargaining chip in financial negotiations This paradigm of vulnerability problematizes adaptation of vulnerable populations towards the international community to access and gain control over already scarce funding, yet, these problematizations are often disconnected from local adaptive realities and needs, leading to financial and institutional support for technological fixes and mega-projects. With this, as in our case, there is (too) little left to support those vulnerable and in need for adaptation to this rapidly changing former lake environment amidst ongoing social and political changes. Consequently, there is neither a search for nor an acknowledgement of alternative solutions such as mobile husbandry but rather a continuation of a dialogue between state and international community, with limited visibility of local established practices and needs. Hence, vulnerability (re)produces narratives that turns project enactors into the key actors and transform the label of vulnerability into a non-tangible "commodity" to allow access to promised funds, while failing to address inequities and inequalities that are a root cause of vulnerabilities, that exclude local actors and their visions and perceptions of adaptation, as we argue in Djoudi and Brockhaus 2016. Instead, we see a process of maladaptation, expressed in persistent discursive practices that polarise

pastoralism and sedentarism and render what is political as technical in terms of peoples' choices of how to respond to climate change.

From BAU to TC

Adaptation policy and finance are and can, at least to some extent, provide new incentives and discourses for a transformational change away from maladaptation and inequality, as seen on this frontier. However, the politics that produce adaptation policy, including science-policy interactions, will need to critically examine underlying assumptions and internal structures and architecture to avoid repeating and reinforcing the business-as-usual practices borne out of colonial legacies.

Case 4. The 4Is, cross-level power inequalities and the integration of climate change adaptation, mitigation and development

The case presented here is about Brazil's and Indonesia's forest frontiers and investigates the integration of mitigation, adaptation and development objectives across three tiers of governance; national, state/province, and municipality/district levels, with the local focus on Mato Grosso and West Kalimantan. Specifically, this case illustrates climate policy processes across levels as they play out on the local forest frontier and which agenda and whose objectives matter. Below, we discuss how information, interests, institutions and ideas shape climate and development policy dynamics among actors across governance levels.

Integration and information gaps across levels of governance

Conceptually, experts advocate integration of the three objectives (mitigation, adaptation and development) in the tropical forest frontier as a way to balance global and local interests and deliver climate change responses, while ensuring local development opportunities and environmental justice (Bustamante et al.

2014; Locatelli et al. 2015;, Nunan et al. 2017). Effective integration ensures that trade-offs are minimised and synergies are implemented (Klein et al. 2005; Ravindranath 2007; Smith et al. 2014; Kongsager et al. 2015; IPCC 2019). However, in the forest and climate domain, achieving integration has largely been elusive (Locatelli et al. 2011; Thuy et al. 2014; Di Gregorio et al. 2017b). One basic challenge relates to information: the lack of expertise and knowledge gaps about climate change adaptation of local livelihoods are quite extensive. This is in part due to the context specific nature of climate change impacts and of vulnerability, which makes them more complex than mitigation. Because climate change impacts and related costs are felt at the local level, climate change adaptation responses will differ across countries, ecosystems, groups and individuals that display distinct vulnerabilities (Denton et al. 2014; Locatelli et al. 2015; Adger 2001). In contrast, climate change mitigation in forests is more straightforward and can be tackled by a limited number of actions that reduce emissions (Locatelli et al. 2015). We found that knowledge gaps were much more extensive for adaptation than mitigation and were unequally distributed across governance levels, with expertise on climate change adaptation primarily located on a national level, when it is most needed on a local level. Information is a major power resource and this lack of local expertise translated in the inability of local governments to effectively demand attention to and effectively address local adaptation needs related to droughts, floods and fires in both forest frontiers, particularly in Indonesia (Di Gregorio et al. 2017b; Di Gregorio et al. 2019).

Global institutions, interests and ideas

Overall, the greatest challenge to effective integration is **politico-economic** (Di Gregorio et al. 2017b; Ellis and Tschakert 2019). In tackling climate change, global, national and local **interests** value such actions differently. Climate change mitigation benefits accrue primarily on a global level and mitigation requires concerted efforts by most countries to be effective (Locatelli et al. 2011; Dolšak and Prakash 2018). Consequently, considerable efforts within global climate change **institutions** focus on ensuring cooperation across countries regarding mitigation action. Because the costs of climate change impact and the benefits of adaptation are carried locally, global climate finance focuses more on mitigation than adaptation efforts,

despite richer countries being responsible for the vast majority of historical emissions that cause climate change impacts in poorer countries (Pielke et al. 2007; Winkler and Dubash 2016). These institutional level impacts were observed in both Brazil and Indonesia and have created their own path-dependencies and lock-ins.

In both Brazil and Indonesia, climate change action is primarily funded through bilateral aid, from Norway, Germany and the UK, while multilateral aid from the World Bank and United Nations agencies is also key in Indonesia. The dependence on the Global North for climate finance led to mitigation action being prioritised over adaptation programs in both countries. Further, **ideas** become power resources through discursive practices. Global climate change discourse has long prioritized mitigation as it focuses on forests almost exclusively as high mitigation potential carbon sinks (Stern 2007). Consequently, climate change adaptation of people living in the forest margins has long been neglected, despite growing concerns related to major impacts, such as more extensive droughts in the Amazon region (Davidson et al. 2012).

National institutions, interests and ideas

Regarding national **institutions**, we found that federalism in Brazil and decentralization arrangements in Indonesia affected power structures across governance levels in the climate change domain. In Brazil federal level actors were most influential, followed by state level actors, who in turn dominated the municipal level. In the state of Mato Grosso, in the Brazilian arc of deforestation, forest governance featured powerful economic livestock and soy agribusiness **interests** supported by national level allies and local landowners' associations. Contraposed were long standing but less well-resourced environmental conservation and climate change mitigation interests (Saito and Azevedo 2016; Di Gregorio et al. 2019). The federal and local environmental agencies monitored conservation, deforestation and forest fires. They were confronted by agribusiness and landowners who resented federal interference, underplayed their contribution to GHG emissions and ignored the environmental justice agenda. Since Bolsonaro's election and the re-emergence of climate change denialist **ideas** and discourses, the resulting withdrawal of federal support for a cross-level

transformative policy alliance crippled mitigation efforts further jeopardizing any integration of climate change and development objectives (Ferrante and Fearnside 2019).

In Indonesia, **institutional** decentralization legacies still provided high autonomy to districts. National actors dominated multi-level power structures, but districts held more influence than intermediate provincial governments. In West Kalimantan agribusiness-led development focusing on oil palm production was strongly supported by all government levels (Di Gregorio et al. 2019). Government actors had internalised the **interests** of large-scale business without the need for them to do any direct lobbying (Cramb and McCarthy 2016). International environmental NGOs led forest-based mitigation efforts in alliance with (minority) conservation **interests** within government, while less powerful civil society organisations and under-resourced environmentally focused state actors called for an improved balance between conservation and development. Still, the local forestry agency that aimed to balance development with conservation strategies adopted the climate change mitigation **discourse** to attract finance to deliver sustainable forest management to support local development, but reducing carbon emissions was not its main goal (Di Gregorio et al. 2019).

From BAU to TC

Global and national power structures favoring climate change mitigation over adaptation translated to a climate change agenda on both Brazil's and Indonesia's forest frontiers that focused nearly exclusively on climate change mitigation. However, this agenda remained largely outcompeted by domestic extractivist approaches to development, resulting in increasing carbon emissions from forests (Di Gregorio et al. 2019; Turubanova et al. 2018). Local climate change adaptation needs were not well understood and demands for climate justice were mainly raised by the least powerful group of local development NGOs and remained neglected in policy and practice impacting negatively marginalised and vulnerable groups. Under these circumstances **transformational change and the required shifts in discursive practices, incentive structures and power relations** can only advance if an alliance emerges between civil society and key reformist government actors willing to push climate change adaptation and provide a vision of an alternative and

sustainable form of local development that caters to local needs and redirects attention from short-term interests of the national treasury and global markets to long-term resilience building in local communities.

Case-specific references

Adger, W.N. 2001. Scales of governance and environmental justice for adaptation and mitigation of climate change. Journal of International Development 13, 7: 921-931.

Albrecht, G., G.M. Sartore, L. Connor, N. Higginbotham, S. Freeman, B. Kelly, H. Stain, A. Tonna, and G. Pollard. 2007. Solastalgia: the distress caused by environmental change. Australasian psychiatry, 15(sup1): S95-S98.

Angelsen, A., and D. Kaimowitz. 1999. Rethinking the causes of deforestation: lessons from economic models. The world bank research observer 14(1):73-98.

Baird, I.G. 2017. Resistance and contingent contestations to large-scale land concessions in southern Laos and northeastern Cambodia. Land 6(1):16.

Bryant, R. L. 1996. Romancing colonial forestry: The discourse of 'forestry as progress' in British Burma. Geographical Journal:169-178.

Bustamante, M., C. Robledo-Abad, R. Harper, C. Mbow, N.H. Ravindranat , F. Sperling, H. Haberl, A.S. Pinto, and P. Smith. 2014. Co-benefits, trade-offs, barriers and policies for greenhouse gas mitigation in the agriculture, forestry and other land use (AFOLU) sector. Global Change Biology 20(10):3270-3290.

Climate Focus. 2016. Progress on the New York Declaration on Forests: Eliminating Deforestation from the Production of Agricultural Commodities–Goal 2 Assessment Report. Prepared by Climate Focus in cooperation with the NYDF Assessment Coalition with support from the Climate and Land Use Alliance and the Tropical Forest Alliance 2020.

Cole R., G. Wong, and I.W. Bong. 2017b. Implications of the ASEAN Economic Community (AEC) for trans-boundary agricultural commodities, forests and smallholder farmers. CIFOR Infobrief no. 178. Bogor, Indonesia: CIFOR.

Cramb, R.A. and, J.F. McCarthy (Eds.). 2016. The oil palm complex: Smallholders, agribusiness and the state in Indonesia and Malaysia. Singapore: NUS Press.

Cuypers, D., T. Geerken, L. Gorissen , A. Lust, G. Peters, J. Karstensen , S. Prieler , G. Fischer, E. Hizsnyik, and H. Van Velthuizen. 2013. The impact of EU consumption on deforestation: Comprehensive analysis of the impact of EU consumption on deforestation. European Union. http://pure.iiasa.ac.at/id/eprint/14868/1/1.%20Report%20analysis%20of%20impact.pdf .

Davidson, E.A., A.C. de Araujo, P. Artaxo, J.K. Balch, I.F. Brown, M.M.C. Bustamante , M.T. Coe, R.S. DeFries, M. Keller, M. Longo, J.W. Munger, W. Schroeder, B.S. Soares-Filho, C.M. Souza, S.C. Wofsy, et al. 2012. The Amazon basin in transition. Nature 481, 7381: 321-328.

De Sy, V., M. Herold, M. Brockhaus, M. Di Gregorio, and R.M. Ochieng. 2018. Information and policy change: Data on drivers can drive change–if used wisely. In Angelsen A, Martius C, De Sy V, Duchelle AE, Larson AM and Pham TT (Eds.). Transforming REDD+: Lessons and new directions. p. 55–68. Bogor, Indonesia: CIFOR.

Denton, F., T.J. Wilbanks, A.C. Abeysinghe, I. Burton, Q. Gao 534 , M.C. Lemos, T. Masui, K.L. O'Brien, K. Warner, et al. 2014. Climate resilient pathways: Adaptation, mitigation, and sustainable development, in: C.B. Field, V.R. Barros, D.J. Dokken, K.J. Mach, M.D. Mastrandrea , T.E. Bilir , M. Chatterjee, K.L. Ebi, Y.O. Estrada, R.C. Genova , B. Girma, E.S. Kissel, A.N. Levy, S. MacCracken, P.R. Mastrandrea, and L.L. White. Ed. Climate Change 2014: Impacts, Adaptation, and Vulnerability. Part A: Global and Sectoral Aspects. Contribution of Working Group II to the Fifth Assessment Report of the Intergovernmental Panel on Climate Change. 1101-1131. Cambridge, United Kingdom and New York, NY: Cambridge University Press.

Dkamela, G. P. 2011. The context of REDD+ in Cameroon: drivers, agents and institutions. Occasional Paper. Bogor, Indonesia: CIFOR.

Dkamela, G., M. Brockhaus, F. Kengoum Djiegni, J. Schure, and S. Assembe Mvondo. 2014. Lessons for REDD+ from Cameroon's past forestry law reform: a political economy analysis. Ecology and Society 19(3):30.

Dolšak, N., and A. Prakash. 2018. The Politics of Climate Change Adaptation. Annual Review of Environment and Resources 43 (1):317-341.

Doolittle, A. 2003. Colliding discourses: Western land laws and native customary rights in North Borneo, 1881-1918. Journal of Southeast Asian Studies 34(1):97-126.

Duncan, R. 1994. Melanesian forestry sector study. International Development Issues No. 36. Canberra, Australia: Australian International Development Assistance Bureau.

Dwisatrio B, Z. Said, A.P. Permatasar, C. Maharani, M. Moeliono, A. Wijaya, A.A. LestarI, J. Yuwono, and T.T. Pham. 2021. The context of REDD+ in Indonesia: Drivers, agents and institutions– 2nd edition. Occasional Paper 216. Bogor, Indonesia: CIFOR.

Ellis, N.R., and P. Tschakert. 2019. Triple-wins as pathways to transformation? A critical review. Geoforum 103: 167-170.

Ferrante, L., and P.M. Fearnside. 2019. Brazil's new president and 'ruralists' threaten Amazonia's environment, traditional peoples and the global climate. Environmental Conservation 46(4): 261-263.

Fox, J., Y. Fujita, D. Ngidang, N. Peluso, L. Potter, N. Sakuntaladewi, J. Sturgeon, and D. Thomas. 2009. Policies, political-economy, and Swidden in Southeast Asia. Human Ecology 37: 305–322.

Galudra, G., and M. Sirait. 2009. A Discourse on Dutch Colonial Forest Policy and Science in Indonesia at the Beginning of the 20th Century. International Forestry Review 11(4): 524–533.

Garrett, R.D., S. Levy, K.M. Carlson, T.A. Gardner, J. Godar, J. Clapp, P. Dauvergne, R. Heilmayr, Y. le Polain de Waroux, B. Ayre, R. Barr, B. Døvre, H.K. Gibbs, S. Hall, S. Lake, J.C. Milder, L.L. Rausch, R. Rivero, X. Rueda, R. Sarsfield, B. Soares-Filho, and N. Villoria. 2019. Criteria for effective zero-deforestation commitments. Global Environmental Change 54: 135-147.

Geist, H., and E. Lambin. 2002. Proximate causes and underlying driving forces of tropical deforestation. BioScience 52: 143–150.

Gonin, A., and D. Gautier. 2015. Shift in herders' territorialities from regional to local scale: the political ecology of pastoral herding in western Burkina Faso. Pastoralism 5(1):7.

Grabs, J., G. Auld, and B. Cashore. 2020. Private regulation, public policy, and the perils of adverse ontological selection. Regulation & Governance.

Gupta, A., I. Boas, and P. Oosterveer. 2020. Transparency in global sustainability governance: to what effect? Journal of Environmental Policy & Planning, 22(1): 84-97.

Heinimann, A., O. Mertz, S. Frolking, A. E. Christensen, K. Hurni, F. Sedano, L. P. Chini, R. Sahajpal, et al. 2017. A global view of shifting cultivation: Recent, current, and future extent. PLoS ONE 12: e0184479.

Heyd, T. and Brooks, N., 2009. Exploring cultural dimensions of adaptation to climate change. In Adger WN, Lorenzoni I, O'Brien KL (Eds.). Adapting to climate change: Thresholds, values, governance. Cambridge University Press: 269-282.

Hrabanski, M. 2017. Private Sector Involvement in the Millennium Ecosystem Assessment: Using a UN platform to promote market-based instruments for ecosystem services. Environmental Policy and Governance, 27(6), 605-618.

Indrarto, G.B., P. MurharjantiJ. Khatarina, I. Pulungan, F. Ivalerina, J. Rahman, M.N. Prana, I.A.P. Resosudarmo, and E. Muharrom. 2012. The context of REDD+ in Indonesia: drivers, agents and institutions. Occasional Paper. Bogor, Indonesia: CIFOR.

IPCC. 2019. Climate Change and Land: an IPCC special report on climate change, desertification, land degradation, sustainable land management, food security, and greenhouse gas fluxes in terrestrial ecosystems. Report, Intergovernmental Panel on Climate Change, Switzerland.

Kaimowitz, D and Angelsen A. 1998. Economic Models of Tropical Deforestation: A Review. Bogor, Indonesia: CIFOR.

Kenney-Lazar, M., M. Dwyer, and C. Hett. 2018a. Turning Land into Capital: Assessing A Decade of Policy in Practice. A Report Commissioned by the Land Information Working Group (LIWG). Vientiane, Laos.

Kenney-Lazar, M., D. Suhardiman, and M.B. Dwyer. 2018b. State spaces of resistance: industrial tree plantations and the struggle for land in Laos. Antipode 50(5): 1290-1310.

Kerkvliet, B.J.T. 2014. Protests over land in Vietnam: Rightful resistance and more. Journal of Vietnamese Studies 9(3) 19-54.

Klein, R.J.T., E.L.F. Schipper, and S. Dessai. 2005. Integrating mitigation and adaptation into climate and development policy: Three research questions. Environmental Science and Policy 8: 579-588.

Koffi, C.K., H. Djoudi, and D. Gautier. 2017. Landscape diversity and associated coping strategies during food shortage periods: evidence from the Sudano-Sahelian region of Burkina Faso. Regional Environmental Change 17(5): 1369-1380.

Kongsager, R., B. Locatelli, and F. Chazarin. 2015. Addressing Climate Change Mitigation and Adaptation Together: A Global Assessment of Agriculture and Forestry Projects. Springer.

Kramarz, T., and Park, S., 2016. Accountability in Global Environmental Governance: A Meaningful Tool for Action? Global Environmental Politics 16(2):1-21.

Lambin, E.F., and T. Thorlakson. 2018. Sustainability standards: Interactions between private actors, civil society, and governments for REDD+ Policymakers. Annual Review of Environment and Resources 43:369-393.

Larsen, R.K., M. Osbeck , E. Dawkins , H. Tuhkanen, H. Nguyen, A. Nugroho, T.A. Gardner, and P. Wolvekamp. 2018. Hybrid governance in agricultural commodity chains: Insights from implementation of ‘No Deforestation, No Peat, No Exploitation’(NDPE) policies in the oil palm industry. Journal of Cleaner Production 183:544-54.

Laxén, J. 2007. Is prosopis a curse or a blessing? Tropical forestry reports. Finland: VITRI, University of Helsinki, 1-199.

Lestrelin, G., J. C. Castella, and J. Bourgoin. 2012. Territorialising Sustainable Development: The Politics of Land-use Planning in Laos. Journal of Contemporary Asia 42: 581–602.

Lestrelin, G., M. Trockenbrodt, K. Phanvilay, S. Thongmanivong, T. Vongvisouk, T. T. Pham, and J. C. Castella. 2013. The context of REDD+ in the Lao People's Democratic Republic: Drivers, agents and institutions. Occasional Paper 92. Bogor, Indonesia: CIFOR.

Locatelli, B., V. Evans, A. Wardell, A. Andrade, and R. Vignola. 2011. Forests and climate change in Latin America: linking adaptation and mitigation. Forests 2(1): 431-450.

Logo Bigombe, P., J.J. Sinang, and Y. Zo'obo. 2020. Les racines coloniales de la gestion des ressources forestières et fauniques au Cameroun. In Batibonank (Ed) Indépendances inachevées en Afrique: Sur les chemins de la reconquête. Yaoundé, Monange.

Mason, M. 2020. Transparency, accountability and empowerment in sustainability governance: a conceptual review. Journal of Environmental Policy & Planning 22(1): 98-111.

May, P. H., M. F. Gebara, L. M. Barcellos, M. Rizek, and B. Millikan. 2016. The context of REDD+ in Brazil: Drivers, actors and institutions, 3rd Edition. Occasional Paper 160. Bogor, Indonesia: CIFOR.

Mertz, O., C. Padoch, J. Fox, R. A. Cramb, S. J. Leisz, N. T. Lam, and T. D. Vien. 2009. Swidden change in Southeast Asia: understanding causes and consequences. Human Ecology, 37(3): 259-264.

Meyfroidt, P., J. Börner, R. Garrett, T. Gardner, J. Godar, K. Kis-Katos, B. S. Soares-Filho, and S. Wunder. 2020. Focus on leakage and spillovers: Informing land-use governance in a tele-coupled world. Environmental Research Letters 15(9): 090202.

Meyfroidt, P., E.F. Lambin, K. H. Erb, and T. W. Hertel. 2013. Globalization of land use: Distant drivers of land change and geographic displacement of land use. Current Opinion in Environmental Sustainability 5: 438-444.

Mikulewicz, M. 2020. Disintegrating labour relations and depoliticized adaptation to climate change in rural São Tomé and Príncipe. Area: 1-9.

Mol, A. 2014. The lost innocence of transparency in environmental politics. In A. Gupta and M. Mason (Eds.), Transparency in global environmental governance, 39–60. Cambridge, MA: MIT Press.

Mpoyi, A. M., F. B. Nyamwoga, F. M. Kabamba, and S. Assembe-Mvondo. 2013. The context of REDD+ in the Democratic Republic of Congo: Drivers, agents and institutions. Occasional Paper 94. CIFOR, Bogor, Indonesia.

Myers, R., D. Intarini, M. T. Sirait, and A. Maryudi. 2017. Claiming the forest: Inclusions and exclusions under Indonesia's 'new' forest policies on customary forests. Land Use Policy 66: 205-213.

Nanhthavong, V., M. Epprecht, C. Hett, J. G. Zaehringer, and P. Messerli. 2020. Poverty trends in villages affected by land-based investments in rural Laos. Applied Geography 124: 102298.

Niamir-Fuller, M. 1999. Managing mobility in African rangelands. In N. McCarthy, B. Swallow, M. Kirk and P. Hazell (Eds.), Property rights, risk and livestock development in Africa, 102-31. Washington, DC: IFPRI.

Nori, M., and I. Scoones. 2019. Pastoralism, uncertainty and resilience: global lessons from the margins. Pastoralism 9(1): 1-7.

Nunan, F. 2017. Making climate compatible development happen. Routledge, Abingdon, UK and New York, NY, USA.

Nunow, A. A. 2000. Pastoralists and markets: livestock commercialization and food security in north-eastern Kenya. People 11: 191.

Pacheco P., H. Bakhtary, M. Camargo, S. Donofrio, I. Drigo and D. Mithöfer. 2018. The private sector: Can zero deforestation commitments save tropical forests? In A. Angelsen, C. Martius, V. De Sy, A. E. Duchelle, A. M. Larson and T. T. Pham (Eds). Transforming REDD+: Lessons and New Directions, 161–173. Bogor, Indonesia: CIFOR.

Painter, T., J. Sumberg, and T. Price. 1994. Your" Terroir" and My 'Action Space': Implications of Differentiation, Mobility and Diversification for the "Approche Terroir" in Sahelian West Africa. Africa: 447-464.

Pasiecznik, N., H. Savenije, C. Van Orshoven, J. Bock, and P. Pacheco . 2017. Key issues: Making zero deforestation commitments work better. ETFRN News 58.

Pattberg, P. H. 2005. The Forest Stewardship Council: Risk and potential of private forest governance. Journal of Environment and Development 14: 356–374.

Pendrill, F., U. M. Persson, J. Godar, T. Kastner, D. Moran, S. Schmidt, and R. Wood. 2019. Agricultural and forestry trade drives large share of tropical deforestation emissions. Global Environmental Change 56.

Pham, T. T., M. Moeliono, M. Brockhaus, D. N. Le, G. Y. Wong, and T. M. Le. 2014. Local preferences and strategies for effective, efficient, and equitable distribution of PES revenues in Vietnam: Lessons for REDD+. Human Ecology 42.

Pham, T. T., M. Moeliono, T. H. Nguyen, H. T. Nguyen, and T. H. Vu. 2019. The context of REDD+ in Vietnam: Drivers, agents and institutions, 2nd edition. Occasional Paper 75. Bogor, Indonesia: CIFOR.

Pielke, R.J., G. Prins, S. Rayner, and D. Sarewitz. 2007. Lifting the taboo on adaptation. Nature 445: 597-598.

Pirard, R., A. Fishman, S. Gnych, K. Obidzinski, and P. Pacheco. 2015. Deforestation-free commitments: The challenge of implementation – An application to Indonesia. Working Paper 181. Bogor, Indonesia: CIFOR.

Pramova, E., B. Locatelli, H. Djoudi, and O. A. Somorin. 2012. Forests and trees for social adaptation to climate variability and change. Wiley Interdisciplinary Reviews: Climate Change 3(6): 581-596.

Rachman, N.F., and M. Siscawati. 2016. Forestry Law, Masyarakat Adat and Struggles for Inclusive Citizenship in Indonesia. In C. Antons (Ed.) Routledge Handbook of Asian Law, 238-263. Routledge.

Ravindranath, N.H. 2007. Mitigation and adaptation synergy in the forest sector. Mitigation and Adaptation Strategies and Global Change 12: 843-853

Reenberg, A. 2012. Insistent dryland narratives: portraits of knowledge about human-environmental interactions in Sahelian environment policy documents. West African Journal of Applied Ecology, 20(1): 97-111.

Royer, S. D. E., M. van Noordwijk, and J. M. Roshetko. 2018. Does community-based forest management in Indonesia devolve social justice or social costs? International Forestry Review 20: 167–180.

Rudel, T., and Roper, J. 1996. Regional patterns and historical trends in tropical deforestation, 1976-1990: A qualitative comparative analysis. Ambio 25(3): 160-166.

Saito, C.H., A.A. Azevedo. 2017. Organic intellectuals: legitimizing agribusiness production in Brazil. International Gramsci Journal 2: 107-132.

Setyowati, A.B. 2020. Governing the ungovernable: contesting and reworking REDD+ in Indonesia. Journal of Political Ecology, 27(1).

Smith, P., M. Bustamante, H. Ahammad, H. Clark, H. Dong,E.A. Elsiddig, H. Haberl,R. Harper,J. House, M. Jafari, O. Masera, C. Mbow, N.H. Ravindranath, C.W. Rice,C. Robledo Abad, A. Romanovskaya, F. Sperling, F. Tubiello . 2014 . Agriculture, forestry and other land use (AFOLU), in: Edenhofer, O., Pichs-Madruga, R., Sokona, Y., Farahani, E., Kadner, S., Seyboth, K., Adler, A., Baum, I., Brunner, S., Eickemeier, P., Kriemann, B., Savolainen, J., Schlömer, S., von Stechow, C., Zwickel, T., and Minx, J.C. (Eds.), Climate change 2014: Mitigation of climate change. Contribution of Working Group III to the fifth assessment report of the Intergovernmental Panel on Climate Change Cambridge University Press, Cambridge, United Kingdom and New York, NY, USA.

Stern, N. 2007. The economics of climate change: The Stern review. Cambridge University Press, Cambridge.

Thuy, P. T., M. Moeliono, B. Locatelli, M. Brockhaus, M. Di Gregorio, and S. Mardiah. 2014. Integration of adaptation and mitigation in climate change and forest policies in Indonesia and Vietnam. Forests 5.

To, P., and W. Dressler. 2019. Rethinking 'success': The politics of payment for forest ecosystem services in Vietnam. Land Use Policy 81: 582–593.

Toulmin C., and K. Brock. 2016. Desertification in the Sahel: Local practice meets global narrative. In: Behnke R., Mortimore M. (Eds.) The End of Desertification? Springer Earth System Sciences. Springer, Berlin, Heidelberg.

Tschakert, P., and R. Tutu. 2010. Solastalgia: Environmentally induced distress and migration among Africa's poor due to climate change. In Environment, Forced Migration and Social Vulnerability, 57-69. Springer, Berlin, Heidelberg.

Turner, M. D. 2011. The new pastoral development paradigm: Engaging the realities of property institutions and livestock mobility in dryland Africa. Society and Natural Resources, 24(5): 469-484.

Turner, M. D. 2017. Livestock mobility and the territorial state: South-Western Niger (1890–1920). Africa 87(3): 578-606

Turner, S., and D. Caouette. 2009. Shifting fields of rural resistance in Southeast Asia. In D. Caouette and S. Turner (Eds). Agrarian Angst and Rural Resistance in Contemporary Southeast Asia. Routledge, Oxford.

Turubanova, S., P.V. Potapov, A. Tyukavina, M.C. Hansen. 2018. Ongoing primary forest loss in Brazil, Democratic Republic of the Congo, and Indonesia. Environmental Research Letters 13(7): 074028.

UNEP. 2009. Ecosystem Management for Improved Human Well-Being in The Lake Faguibine System: Conflict Mitigation and Adaptation to Climate Change. http://www.unep.org/pdf/Lake-Faguibine.pdf.

Vandergeest, P., and N. L. Peluso. 2006. Empires of forestry: Professional forestry and state power in Southeast Asia, Part 1. Environment and History, 12(1): 31-64.

Weatherley-Singh, J., and A. Gupta. 2015. Drivers of deforestation and REDD+ benefit-sharing: A meta-analysis of the (missing) link. Environmental Science and Policy 54:97-105.

Winkler, H., and N.K. Dubash. 2016. Who determines transformational change in development and climate finance? Climate Policy 16(6): 783-791.
